# Therapeutic Potential of Saffron Extract in Mild Depression: A Study of Its Role on Anhedonia in Rats and Humans

**DOI:** 10.1002/ptr.8424

**Published:** 2025-01-04

**Authors:** Eleonora Corridori, Sara Salviati, Maria Graziella Demontis, Pamela Vignolini, Chiara Vita, Andrea Fagiolini, Alessandro Cuomo, Pietro Carmellini, Carla Gambarana, Simona Scheggi

**Affiliations:** ^1^ Department of Molecular and Developmental Medicine, School of Medicine University of Siena, Polo Universitario San Miniato Siena Italy; ^2^ Laboratorio Phytolab‐DiSIA University of Florence Florence Italy; ^3^ PIN‐QuMAP Polo Universitario di Prato Prato Italy; ^4^ Division of Psychiatry, Department of Molecular and Developmental Medicine, School of Medicine University of Siena Siena Italy

**Keywords:** brain‐derived neurotrophic factor (BDNF), dopamine, Montgomery–Åsberg depression rating scale (MADRS), nucleus accumbens, progressive ratio, stress

## Abstract

Drugs generally used in major depressive disorder are considered inappropriate for the more common milder forms. The efficacy of saffron extracts has been demonstrated in mild to moderate depression and in preclinical models of depression. However, evidence of saffron activity on reduced hedonic responsiveness and motivational anhedonia is limited. Since dopamine transmission dysfunctions are crucially involved in anhedonia and saffron seems to positively modulate dopamine release, we studied the potential antidepressant and anti‐anhedonic effects of a standardized formulation of saffron extract in preclinical models of anhedonia‐like behaviors, and patients diagnosed with unipolar or bipolar depression. We tested saffron activity in a rat model of stress‐induced motivational anhedonia using sucrose self‐administration protocols and investigated the molecular underpinnings of this effect focusing on DARPP‐32 phosphorylation pattern in response to a reinforcer and BDNF–TrkB signaling, in the nucleus accumbens and medial prefrontal cortex. In parallel, with a pilot double‐blind placebo‐controlled study we investigated whether saffron add‐on therapy reduced symptoms of depression and anhedonia, measured by the Montgomery–Åsberg Depression Rating Scale. Repeated saffron administration restored motivation and reactivity to reward‐associated cues in anhedonic rats, likely modulating dopaminergic transmission and BDNF–TrkB signaling. In depressed patients, an 8‐week saffron add‐on therapy induced a global improvement in depressive symptoms and a significant reduction in anhedonia. The study supports a pro‐motivational effect of saffron and suggests a potentially useful saffron‐based augmentation strategy in anhedonic patients, albeit with limitations due to small sample size and short trial duration.

## Graphical abstract

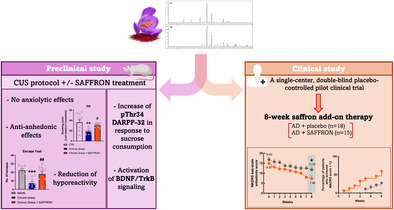

**Abbreviations**
ANH5: MADRS 5‐item anhedonia subscale
BDNF: brain‐derived neurotrophic factor
BP: breaking point
DARPP‐32: dopamine and cAMP‐regulated phosphoprotein, Mr 32 kDa
FR: fixed ratio
MADRS: montgomery–Åsberg depression rating scale
mPFC: medial prefrontal cortex
NAc: nucleus accumbens
PKA: protein kinase A
PR: progressive ratio
SPT: sucrose preference test
SSA: sucrose self‐administration
TrkB: tyrosine receptor kinase B

## 1 Introduction

Mild depression has received increasing attention being twice as common as major depressive disorder and highly prevalent in young people (Fergusson et al. 2005; Carrellas, Biederman, and Uchida 2017; Noyes et al. 2022). For these milder forms, drugs generally used in major depression are considered inappropriate, particularly due to their side effects that reduce adherence to therapy (Naber and Bullinger 2018). Therefore, research in this field is committed to finding novel therapeutic approaches for patients suffering from mild to moderate depression. Among the herbal compounds proposed, saffron extracts, obtained from the dried stigmas of *Crocus sativus L*., demonstrated efficacy in mild to moderate depression (Lopresti and Drummond 2014; Tóth et al. 2019). Saffron, largely used as a spice in cooking, is included in the European Pharmacopoeias, and is used in traditional medicine for its various healing properties, being endowed with a favorable safety profile. Indeed, studies in rodents demonstrated virtually null acute toxicity for the extracts and their components, except for extremely high doses (Bostan, Mehri, and Hosseinzadeh 2017). Four major bioactive compounds have been identified: crocins, crocetin, picrocrocin, and safranal. Randomized, controlled clinical trials and preclinical studies suggest that saffron is an effective treatment, or add‐on treatment, for depression (Orio et al. 2020; Tóth et al. 2019). Moreover, different preclinical studies that examined the potential antidepressant and anxiolytic properties of saffron total extract and/or crocin indicate a correlation with a positive modulation of dopamine and serotonin concentrations in several brain regions, among different possible mechanisms of action (Ettehadi et al. 2013; Monchaux De Oliveira et al. 2021). In light of these modulatory effects, as dopaminergic transmission in the cortico‐mesolimbic circuit plays a relevant role in motivational responsiveness to hedonic stimuli, we hypothesized that saffron may be useful for the treatment of conditions characterized by reduced hedonic responsiveness and motivational anhedonia.

Anhedonia is a frequent symptom of depressive disorders associated with decreased quality of life and is considered a risk factor for the development of severe symptoms, treatment resistance and a chronic illness course (Vanderlind et al. 2020; Lindahl et al. 2023). Moreover, individuals with bipolar disorders experience depressive episodes of high frequency, duration, and chronicity, often with significant levels of anhedonia (Dev et al. 2024; Kupka et al. 2007; Machado‐Vieira et al. 2023). Thus, the identification of treatments that effectively target the domain of reduced motivation and anhedonia in mood disorders is of high clinical relevance. Since the effect of saffron on this domain was not yet specifically investigated, we focused the study on motivational anhedonia and examined the activity of a standardized formulation of freeze–dried extract of saffron farmed in Tuscany in: (i) preclinical models of depressive‐like behaviors in rats and (ii) patients diagnosed with mild unipolar or bipolar depression.

To this end, in the preclinical study, in order to evaluate the anti‐anhedonic effect of saffron we used a reinforcement paradigm, sucrose self‐administration (SSA) protocols, in a rat model of stress‐induced motivational anhedonia (Scheggi et al. 2018). Next, the molecular underpinnings of saffron pro‐motivational effect were investigated. The dopaminergic mesolimbic pathway is involved in motivation toward relevant stimuli (Treadway, Cooper, and Miller 2019) and the dopaminergic response to positive stimuli, characterized by dopamine D1 receptor/Protein Kinase A (PKA)/Dopamine and cAMP‐regulated phosphoprotein Mr. 32 kDa (DARPP‐32) signaling in the nucleus accumbens (NAc) and medial prefrontal cortex (mPFC), is dramatically blunted in anhedonia and is restored by compounds endowed with anti‐anhedonic properties (Scheggi, De Montis, and Gambarana 2018). Thus, we examined whether saffron administration restored the DARPP‐32 phosphorylation pattern in response to a rewarding stimulus in these brain regions. Moreover, since the consequences on dopaminergic neurons of chronic exposure to unavoidable stress likely do not only result in a weakening of reward‐related signaling, we analyzed brain‐derived neurotrophic factor (BDNF)‐tyrosine receptor kinase B (TrkB) signaling in the same brain regions. Indeed, several studies implicated BDNF in the antidepressant effects of saffron (Ghasemi et al. 2015; Mohammadi et al. 2023), and the BDNF–TrkB pathway activation in the NAc has been associated with recovery from emotional deficits (Li et al. 2023). TrkB has two different isoforms, the full‐length TrkB that contains a tyrosine kinase domain and autophosphorylates at tyrosine 515 and 816 upon BDNF binding (TrkB‐FL), and the truncated isoform (TrkB‐T), which lacks the tyrosine kinase domain (Minichiello 2009). We focused on phosphorylation at tyrosine 816, which is considered an index of activation of BDNF downstream signaling, critical for activity‐dependent neuronal plasticity (Enkavi et al. 2024).

In parallel, as anhedonia is a specific clinical dimension that characterizes treatment‐resistant depression in unipolar and bipolar patients (Kelly, Freeman, and Schumacher 2022), an 8‐week, double‐blind placebo‐controlled study was conducted in patients with a current depressive episode diagnosed with unipolar or bipolar mood disorder to investigate whether saffron augmentation reduced symptoms of depression and anhedonia, measured by the clinicians‐rated Montgomery–Åsberg depression rating scale (MADRS). Bipolar patients were enrolled in the study as saffron broad effects on neurotransmitter systems and its safety profile make it a reasonable option for bipolar depression despite the absence of bipolar‐specific preclinical studies (Kell et al. 2017). The urgent need for novel, well‐tolerated treatments in bipolar depression justifies the exploration of saffron as an adjunctive therapy, particularly when other treatments have proven insufficient.

## 2 Material and Methods

### 2.1 Plant Material

Saffron samples originating from the Montalcino cultivation area, in Tuscany, Italy, were provided by Pura Crocus (Siena, Italy) and were classified as category I according to ISO 3632‐1/1993 and ISO 3632‐2/2010, European Food Regulation, relative to crocin, picrocrocin, and safranal content and absence of additives and pesticides.

Dried stigmas were standardized for the content of main components (Phytolab‐Pharmaceutical, Cosmetic, Food supplement Technology and Analysis, University of Florence, Italy) using high‐performance liquid chromatography (HPLC) with a diode array detector (DAD).

### 2.2 HPLC/DAD/MS Analysis

Saffron stigmas were dried at 40°C, extracted with 70% ethanol, exposed to freeze–dry procedure and then analyzed by an HP 1260 liquid chromatograph equipped with a DAD detector and an atmospheric pressure ionization/electrospray ionization (API/ESI) interface (Agilent Technologies, Palo Alto, CA, USA) as detailed in Masi et al. (2016).

### 2.3 Animals

Experiments were carried out on male Sprague–Dawley rats (Charles River, Calco, Italy), 9–10 weeks old when the experimental procedures began. Rats were group‐housed in an environment maintained at a constant temperature (21°C–24°C) and humidity (40%–60%) with free access to food and water, and a 12 h reverse light/dark cycle. Rats were allowed 10 days of habituation to the animal colony, to handling and to the test rooms before the beginning of experimental procedures. Animal care and experimental procedures were in accordance with the European and Italian legislation on the use and care of laboratory animals (EU Directive 2010/63, and D.L. 2014/26). Experimental protocols were approved by the University of Siena Animal Welfare Body and by the Italian Ministry of Health (Authorization N. 874/2021‐PR).

### 2.4 Elevated Plus Maze (EPM) Test

Exposure to unavoidable stressors induces in rodents an increase in anxious‐like behaviors (Padovan and Guimarães 2000), and anxiety is often comorbid with depression and, in general, mood disorders (Saha et al. 2021). Thus, it was of interest to assess the possible effects of the extract on anxiety‐related behaviors, as an anxiolytic activity could contribute to improve performance in the shock‐escape test. To evaluate a potential anxiolytic effect, we used the EPM test that lasts 5 min (Pellow et al. 1985). Rats were individually placed in the central platform of the maze and the time spent in the open and closed arms during the 5 min test was video‐recorded. The percentage of time spent in open or closed arms was calculated by the formula: (time in open or closed arms/time in open + closed arms) × 100. Data were analyzed by trained experimenters, blinded to group assignment.

### 2.5 Induction of Acute Escape Deficit

An escape deficit was induced by exposure to an unavoidable stress session, as described (Gambarana et al. 2001; Scheggi, Pelliccia, et al. 2018). Rats were immobilized with a flexible wire‐net and exposed to tail shocks (1 mA × 5 s, 1 every 30 s for 50 min). Twenty‐four hours later, they were exposed to an avoidable stress session: the shock‐escape test. The criterion for defining the escape deficit condition was a score of 0–6 escapes out of 30 trials (Scheggi, Pelliccia, et al. 2018). A group of rats not exposed to the unavoidable stress session was only exposed to the shock‐escape test to provide a within experiment measure of the baseline reactivity to the test (Naive). The number of escapes was recorded by an experimenter blinded to group assignment.

### 2.6 Chronic Stress Protocol

Rats underwent the unavoidable stress session and the shock‐escape test to assess the development of escape deficit, as described above. Beginning 48 h after the escape test, on alternate days rats were exposed for 10 min to unavoidable stressors: (a) tail shocks of 1 mA × 5 s, 1 every 30 s or (b) immobilization on a flexible wire‐net restrainer, in order to maintain the escape deficit condition (Gambarana et al. 2001; Scheggi, Pelliccia, et al. 2018).

### 2.7 Sucrose Self‐Administration (SSA)

Self‐administration experiments were conducted according to Scheggi, Pelliccia, et al. (2018) in operant chambers (MED Associates Inc., St. Albans, VT, USA) provided with two response levers: the active lever delivered a sucrose pellet (unflavored dustless precision pellets 45 mg, Bio‐Serv, Frenchtown, NJ, USA) into the food receptacle, while the inactive lever produced no programmed result. Experimental events were scheduled, and data were recorded using the MED Associates software (MED Associates Inc.). In all the sessions, rats were non‐food deprived, that is they had free access to standard food pellets before and after the self‐administration sessions. Rats in the control and chronic stress‐exposed groups were trained using the fixed ratio (FR) 1, FR5 and then progressive ratio (PR) schedules. FR1 and FR5 sessions are scheduled to last 15 min, whereas PR sessions, in which the number of responses required to receive a sucrose pellet was progressively increased with a step size of 3 (PR3), ended when 5 min elapsed without a response (breaking point, BP). BP was defined as the number of lever presses in the final completed ratio. The criterion for appetitive motivation deficit, induced by exposure to the chronic stress protocol, was a lever‐pressing rate lower than 60% of the control group rate in FR1 and FR5 schedules (Scheggi, Pelliccia, et al. 2018). Rats in the chronic stress group were exposed to stress sessions in the afternoon, 3–4 h after the end of self‐administration sessions.

### 2.8 Immunoblotting

The mPFC and NAc were excised from the slices that had been identified using the Atlas of Rat Brain corresponding to plates 7–9 and 10–12, respectively (Paxinos and Watson 2007) and immunoblotting was performed as detailed in Scheggi et al. (2020). Briefly, brain tissues were sonicated in 10 volumes of RIPA buffer containing protease inhibitor cocktail (Sigma‐Aldrich), 50 mM sodium fluoride (NaF), 4 mM phenylmethylsulfonyl fluoride (PMSF), then centrifuged at 12,000 × g for 10 min at 4°C to remove cell debris.

The samples, containing 30 μg of total protein, were loaded on precast polyacrylamide gels (4%–15% Criterion TGX Stain‐Free Precast Gel; Bio‐Rad Laboratories) and separated by electrophoresis (SDS‐PAGE) at 130 V for 60–90 min.

Subsequently, the proteins were transferred to a nitrocellulose membrane using the Trans‐Blot Turbo Transfer system (Bio‐Rad Laboratories). The membranes were then blocked for 2 h at room temperature with 3% BSA (Sigma‐Aldrich) in TRIS‐buffered saline (20 mM Tris Base, 150 mM NaCl) containing 0.1% Tween 20, followed by overnight incubation with primary antibodies at 4°C.

The membranes were probed with the following antibodies: anti‐DARPP‐32 (Cell Signaling Technology, Beverly, MA, USA, #2302), anti‐phospho‐Thr34 DARPP‐32 (PhosphoSolutions, Aurora, CO, USA, #p1025‐34), anti‐BDNF (Abcam, ab226843), anti‐TrkB (Cell Signaling, #4603S), anti‐phospho‐TrkB (Millipore, #ABN1381), and anti‐β‐actin (Invitrogen, MA5‐11869).

Following three 10‐min washes, the membranes were incubated with HRP‐conjugated secondary antibodies. Antibody binding was detected using Clarity ECL substrate (Bio‐Rad Laboratories). Band intensities were quantified using the Image Lab software and Gel Doc XRS+ system. Phospho‐protein levels were normalized to those of total unphosphorylated protein, while total protein levels were normalized to β‐actin levels. The values, expressed in arbitrary units, were then calculated as a percentage of the CTR group.

### 2.9 Drugs and Chemicals

The freeze–dried saffron extract was prepared daily in deionized/distilled water and administered i.p. at the dose of 20 or 40 mg/kg/die, in a final volume of 2 mL/kg rat body weight (Ghadrdoost et al. 2011). Control groups received an intraperitoneal (i.p.) administration of saline solution, at the volume of 2 mL/kg rat body weight. Diazepam was purchased from Tocris Bioscience (Bristol, UK) and was suspended in distilled water with 0.3% Tween 80 (vehicle) and administered i.p. in a final volume of 1 mL/kg (Harada et al. 2006). Authentic standards were purchased from Sigma‐Aldrich (St. Louis, USA) or Extrasynthèse S.A. (Lyon, France).

## 3 Experimental Protocols

### 3.1 Experimental Protocol 1

#### 3.1.1 Effects of Saffron Extract on Anxiety‐Like Behaviors in the EPM Test

**FIGURE 1:**
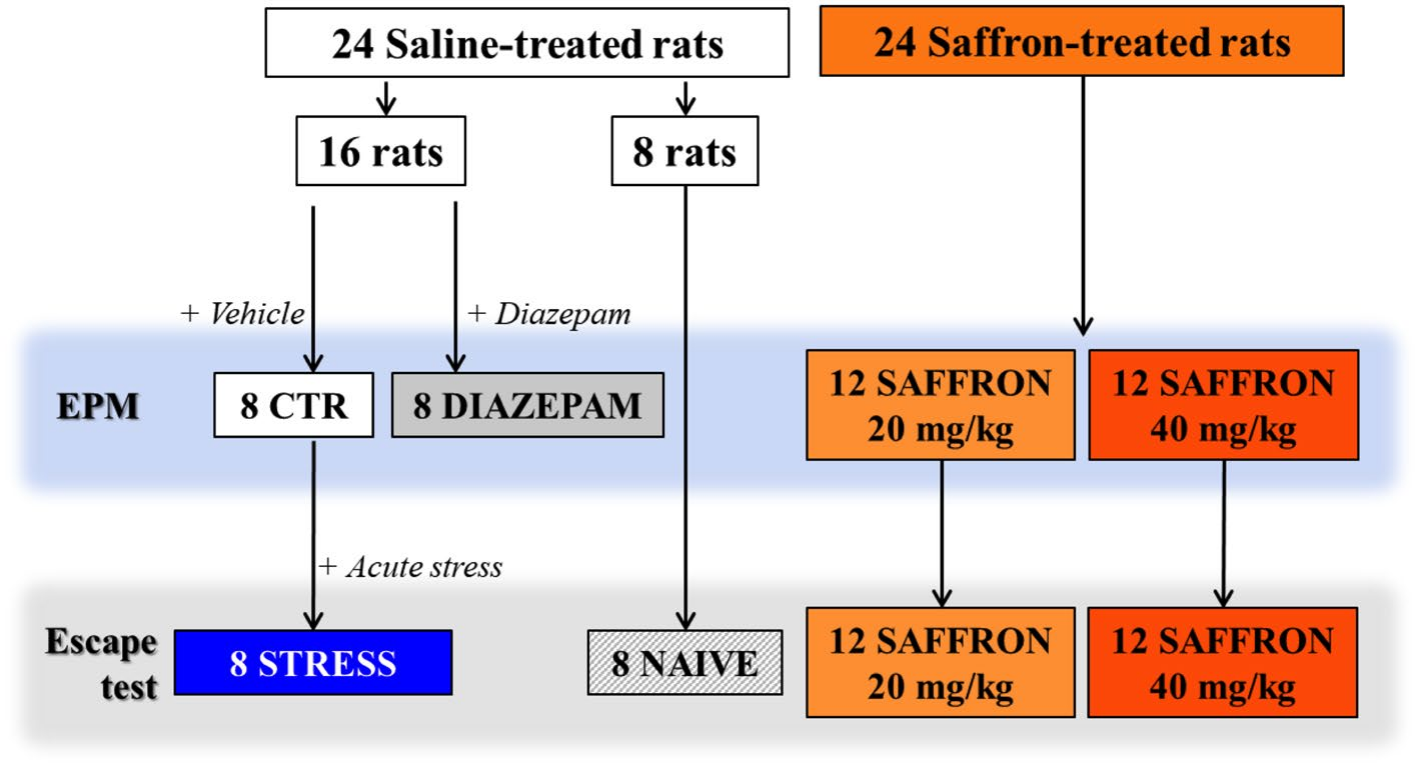
Schematic representation of treatments, group and subgroup assignment in the experimental protocol 1.

In order to evaluate whether long‐term *Crocus sativus L*. extract administration decreased the expression of anxious‐like behaviors, rats (*n* = 48) were divided into three groups that received daily i.p. administration of saline solution (*n* = 24) or saffron extract (20 mg/kg, *n* = 12 or 40 mg/kg, *n* = 12). On the 21st treatment day, the EPM test was performed using: 16 saline‐treated rats and 24 saffron‐treated rats (SAFFRON 20 mg/kg and SAFFRON 40 mg/kg). Thirty min before the test, the saffron‐treated rats and 8 saline‐treated rats (CTR group) received, an i.p. injection of vehicle (distilled water with 0.3% Tween 80) in the volume of 1 mL/kg, while the remaining 8 rats from the saline group received an i.p. injection of diazepam (1 mg/kg) (Diazepam group). Diazepam‐treated rats represent the positive control group for anxiolytic activity and were not used in further experiments. The 8 CTR rats and the saffron‐treated rats continued treatment (Figure 1).

#### 3.1.2 Effects of Repeated Treatment With Saffron Extract on the Prevention of the Escape Deficit Induced by Exposure to Acute Unavoidable Stress

In order to evaluate the possible protective effect of the extract on the development of depressive‐like behaviors induced by exposure to acute unavoidable stress, on day 23, the saline‐ and saffron‐treated rats tested in the EPM test on day 21 were exposed to the unavoidable stress session (pretest) and 24 h later (day 24) to the shock‐escape test. In addition, 8 saline‐treated rats were not exposed to the pretest and were tested as the control group for reactivity to avoidable stress in the shock‐escape test (NAIVE) (Figure 1). This was a crucial experiment preliminary to the assessment of saffron ability to revert the behavioral modifications induced by rats' exposure to a more invasive chronic stress protocol.

Based on the results of this experiment, we selected the higher dose of the extract (40 mg/kg) to be used in the following experiments.

### 3.2 Experimental Protocol 2 Effects of Saffron Extract on Motivational Anhedonia Induced by Exposure to the Chronic Unavoidable Stress Protocol

In order to assess the potential effects of long‐term saffron administration on motivational anhedonia and escape deficit in rats exposed to a chronic unavoidable stress protocol, in a preliminary experiment we investigated whether saffron administration per se affected the acquisition of SSA. After 1 week of treatment, saline‐treated control rats (*n* = 5) and saffron‐treated rats (*n* = 6) were trained in SSA protocols (FR1, FR5 and PR3), while continuing treatment. Thus, the BP was evaluated in rats exposed to treatment for about 23–25 days, considering that FR1 and FR5 sessions last approximately 6–7 and 5–6 days are necessary to perform PR. Long‐term saffron treatment did not induce gross behavioral modifications and did not affect motor activity or performance in the self‐administration protocols, indicating that the extract did not modify cognitive and learning abilities or reward threshold. We then proceeded to evaluate the effects of the extract on the motivational deficits induced by chronic unavoidable stress exposure.

A total of 38 rats were used in this experiment: 20 rats (Chronic stress group) were exposed to the sequence of unavoidable stress session (pretest, day 1) and shock‐escape test (day 2), 8 rats (NAIVE) were only exposed to the shock‐escape test, and 10 rats (control group, CTR) were exposed neither to the unavoidable stress session nor to the shock‐escape test. To maintain the depressive‐like phenotype, the Chronic stress group was exposed to the unavoidable stress protocol until the end of the experiment. On day 14, when the Chronic stress group had already been exposed to stress sessions for 12 days, all rats began the training for SSA (FR1 and FR5 protocols). On day 28, after the fifth FR5 session, treatments began: the CTR group (*n* = 10) received saline; rats in the Chronic stress group were divided into a saline‐treated group (Chronic stress, *n* = 10) and a saffron‐treated group (Chronic stress + SAFFRON, *n* = 10). After 10 days of saline or saffron administration, all rats, exposed or not to stress sessions, resumed training in the SSA protocols (FR5 and PR3), while continuing saline or saffron treatment and stress exposure.

Two days after the end of the SSA experiment, rats in the Chronic stress, Chronic stress + SAFFRON, and NAIVE groups were exposed to the escape test, 18 h after the last treatment.

### 3.3 Experimental Protocol 3 Effects of Saffron Extract on Neurochemical Modifications Induced by Chronic Unavoidable Stress Exposure

We used the same design as in the previous experiment, with the exception of the SSA protocol, in order to study the neurochemical underpinnings of saffron anti‐anhedonic effects without the possible confounding effects of SSA training. Thus, 32 rats were exposed to the unavoidable stress protocol (Chronic stress) and 16 rats were not exposed to the unavoidable stress protocol, control group (CTR). After 12 days of exposure to the stress protocol or not, treatments began for 14 days with saline (CTR, *n* = 16, and Chronic stress, *n* = 16) or saffron (Chronic stress + SAFFRON, *n* = 16). Then, 18 h after the last treatment, rats in each group were subdivided into two subgroups, one sacrificed at time 0 (baseline, *n* = 8/group) and the other 30 min after the consumption of sucrose pellets (*n* = 8/group), to analyze possible modifications in the dopamine D1 receptor‐PKA‐DARPP‐32 pathway in response to the hedonic stimulus. The BDNF/TrkB signaling was examined only in the subgroups of rats sacrificed at baseline. The time point for sacrifice was chosen because the previous experiment showed that around this time, after 10 days of treatment alone and 3–5 days of treatment plus FR5 training, saffron started to show its anti‐anhedonic effects.

### 3.4 Clinical Study

#### 3.4.1 Design

An 8‐week, double‐blind, placebo‐controlled pilot study was conducted at the Division of Psychiatry, University of Siena Medical Center (AOUS), to evaluate the effect of saffron as an add‐on therapy to antidepressants (AD) in patients with mild to moderate depression. Treatment was assigned using a 1:1 randomization ratio. The sample size was calculated based on a power analysis to detect a clinically significant change in the MADRS. Participants were randomized into 2 study arms: one arm (20 subjects) received antidepressant therapy + saffron 30 mg/die (AD + SAFFRON) and the other arm (20 subjects) received antidepressant therapy + placebo (AD + placebo) for 8 weeks.

Saffron extract or placebo was provided in gelatin capsules administered twice a day, 2 capsules for each administration. Saffron capsules contained 7.5 mg of extract, for a daily dose of 30 mg, according to (Akhondzadeh et al. 2005); placebo capsules contained the same excipients as the saffron capsules, except the extract that was substituted by 7.5 mg of calcium phosphate (Table S1).

Overall depression severity was assessed at baseline and weekly for 8 weeks using the MADRS total score by an independent psychiatrist who conducted the clinical interviews. Anhedonia was assessed using the MADRS 5‐item Anhedonia Subscale (ANH5) score, which is based on the following items: (1 obvious sadness; 2 reported sadness, 6 difficulty concentrating, 7 fatigue, 8 inability to feel; McIntyre, Loft, and Christensen 2021). The reduction in MADRS total score at week 8 was the primary efficacy endpoint, with a clinically relevant response defined as a 50% reduction in MADRS total score.

All participants gave written informed consent, and the study was approved by the Institutional Review Board‐Ethics Committee of the University of Siena (protocol code: NAT‐DEP‐ZAFF).

#### 3.4.2 Patients

Eligible subjects aged 18 years or older, had major depressive disorder (MDD) or bipolar disorder (BD) with a current depressive episode. Subjects were referred to the study after a partial response to an antidepressant, suggesting that the current and partially effective antidepressant should not be discontinued, but could benefit from augmentation. Inclusion criteria were (1) a MADRS score > 16 and (2) the absence of psychiatric comorbidity. Patients with gastrointestinal or hepatic diseases, that may affect saffron absorption, were excluded (Figure 1).

### 3.5 Statistical Analysis

Statistical analyses were performed using Prism GraphPad Software 8.0. Multiple ANOVAs as appropriate were performed as described in figure legends; post hoc testing using Sidak's or Dunnett's multiple comparison test was used to compare all pairs of mean or each condition to a control group, respectively. To compare the effect of AD + SAFFRON versus AD + placebo at the endpoint, data were calculated using the independent samples *t* test. The log‐rank test was used to test the difference between the times required to achieve a MADRS score < 12. Data are presented as mean ± SEM. Significance was set at *p* < 0.05. All statistical details are provided in Table S2.

## 4 Results

### 4.1 Qualitative and Quantitative Analysis of Saffron Freeze–Dried Stigmas Hydroalcoholic Extract

**FIGURE 2:**
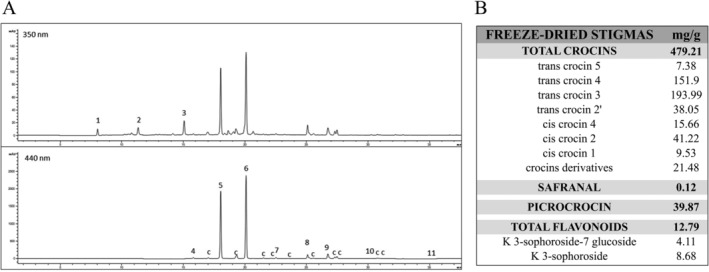
Qualitative and quantitative analysis of saffron freeze–dried stigmas hydroalcoholic extract. (A) Chromatographic profile, acquired at 440 and 350 nm by HPLC‐DAD, of a hydroalcoholic extract from saffron stigmas at relative maximum absorbance of crocins and flavonoids. Identified compounds: (1) kaempferol‐3‐sophoroside‐7‐glucoside, (2) picrocrocin, (3) kaempferol‐3‐sophoroside, (4) trans‐crocin 5, (5) trans‐crocin 4, (6) trans‐crocin 3, (7) trans‐crocin 2′, (8) cis crocin 4, (9) cis crocin 2, (10) cis crocin 1, (11) safranal, c = crocin derivative. (B) Quali‐quantitative analysis of freeze–dried stigmas. Data are expressed as mg/g dry sample. Data are the mean of three determinations (standard deviation 5%). K = kaempferol.

The chromatographic profile, acquired at 440 and 350 nm by HPLC‐DAD, of saffron freeze–dried hydroalcoholic extract identified the compounds listed in Figure 2A. Their qualitative and quantitative analysis are shown in Figure 2B. The total content of crocins was 479.21 mg/g; trans‐crocin 4 and trans‐crocin 3 were the main crocin derivatives present (Vignolini et al. 2008). Among flavonoids, kaempferol‐3‐O‐sophoroside was the main compound.

### 4.2 Effects of Saffron Extract on Anxiety‐Like Behaviors in the EPM Test

**FIGURE 3:**
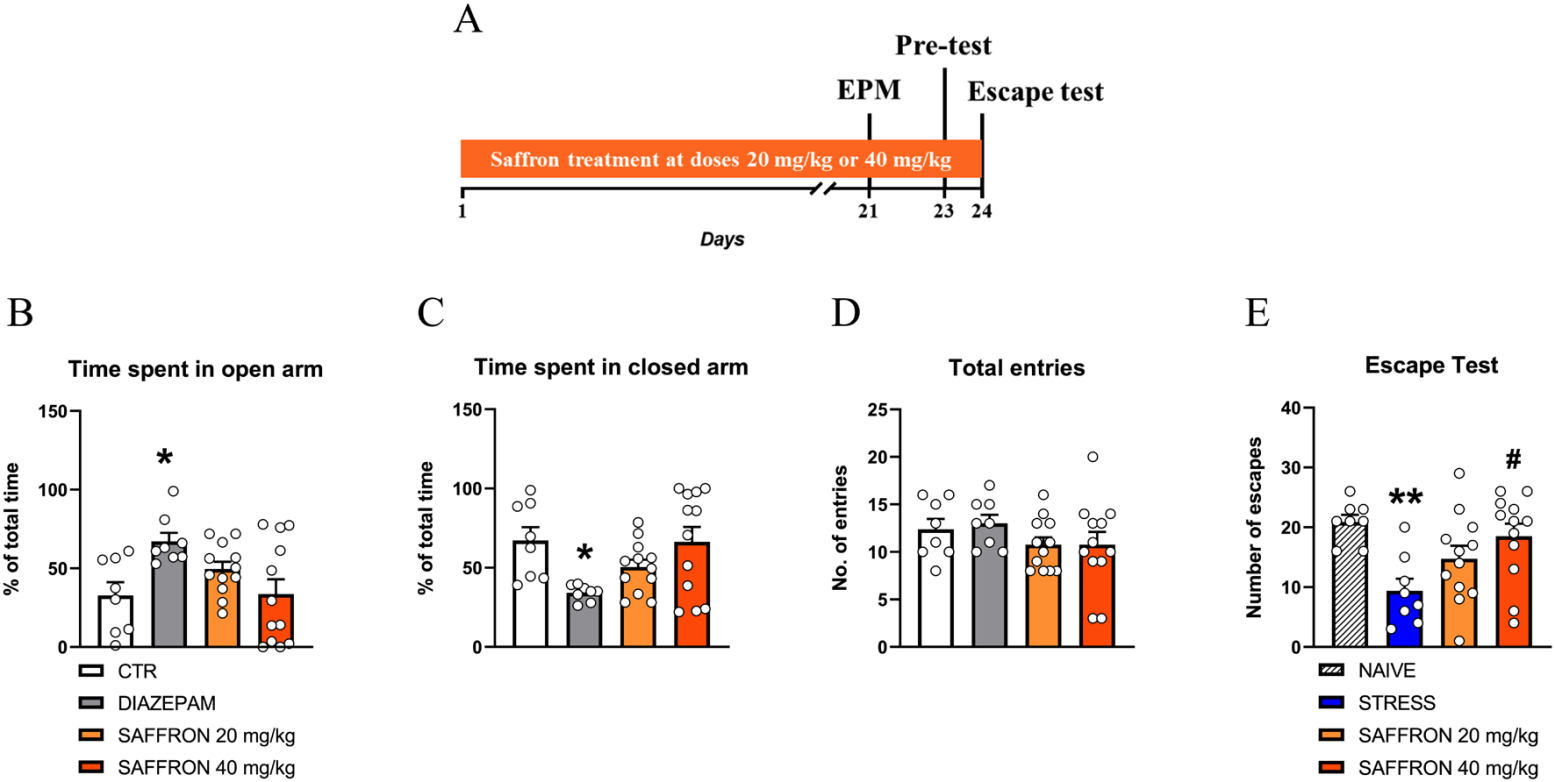
Repeated saffron administration did not show anxiolytic effects and prevented the escape deficit induced by unavoidable stress. (A) Outline of the experiment. (B–D) Time spent in the open (B) or closed arm (C), or overall locomotion (D). Diazepam‐treated rats (1 mg/kg, i.p. 30 min before the test) were used as the positive control group. One‐way ANOVA, (B): *F*_3,36_ = 4.136, *p* < 0.05; (C): *F*_3,36_ = 4.177, *p* < 0.05; (D): *F*_3,36_ = 1.029, *p* = 0.3912. Post hoc comparisons: *p < 0.05 diazepam‐treated rats versus CTR group, Dunnett's multiple comparisons test. (E) Number of escapes, One‐way ANOVA, *F*_3,36_ = 5.020, *p* < 0.01. Post hoc comparisons: **p < 0.01 STRESS versus the NAIVE group; ^#^ *p* < 0.05 SAFFRON 40 mg/kg versus the STRESS group, Dunnett's multiple comparisons test.

This experiment aimed to evaluate whether the saffron extract was endowed with anxiolytic activity (Figure 3A). As depression and anxiety are distinct but closely related phenomena, it was relevant to distinguish an anxiolytic effect in rats not yet exposed to unavoidable stress from the ability to improve coping strategies in rats exposed to stressful cues.

Saffron, at both doses, did not modify the responses in the EPM test compared to the CTR group. Diazepam‐treated rats showed an increase in the time spent in the open arms (*p* < 0.05) and a decrease in the time spent in the closed arms (*p* < 0.05) in comparison to the vehicle‐treated rats, CTR group (Figure 3B,C). Furthermore, the number of total arm entries was similar between groups, suggesting that long‐term administration of the extract did not affect locomotor activity (Figure 3D).

### 4.3 Effects of Repeated Treatment With Saffron Extract on the Prevention of the Escape Deficit Induced by Exposure to Acute Unavoidable Stress

This experiment aimed to evaluate whether the saffron extract prevented the escape deficit induced by acute exposure to unavoidable stress (Figure 3A).

In the escape test, rats never exposed to unavoidable stress (NAIVE) showed normal responsiveness to avoidable noxious stimuli while saline‐treated rats exposed to the pretest session (STRESS) developed a clear‐cut escape deficit (STRESS vs. NAIVE, *p* < 0.01). The administration of saffron extract was effective in preventing the escape deficit at the 40 mg/kg dose (vs STRESS, *p* < 0.05), while at the 20 mg/kg dose only partially increased the reactivity to avoidable nociceptive stimuli, (Figure 3E). Thus, in the following experiments only the 40 mg/kg dose was used.

This was a necessary preliminary experiment in order to step up to the assessment of saffron ability to revert the behavioral modifications induced by exposure to a more invasive chronic stress protocol.

### 4.4 Repeated Treatment With Saffron Extract Rescued Motivational Anhedonia Induced by Exposure to a Chronic Unavoidable Stress Protocol

In order to further assess the potential antidepressant activity of saffron extract, we exposed rats to chronic unavoidable stress and studied saffron ability to reverse motivational anhedonia‐like behavior and escape deficit.

In a preliminary experiment, aimed at evaluating whether saffron affected the performance in SSA protocols, we observed that saffron did not induce gross behavioral modifications and did not affect motor activity, nor did modify SSA acquisition. Responding under FR1, FR5 and PR3 schedules was similar in the CTR and SAFFRON groups (Figure S1).

**FIGURE 4:**
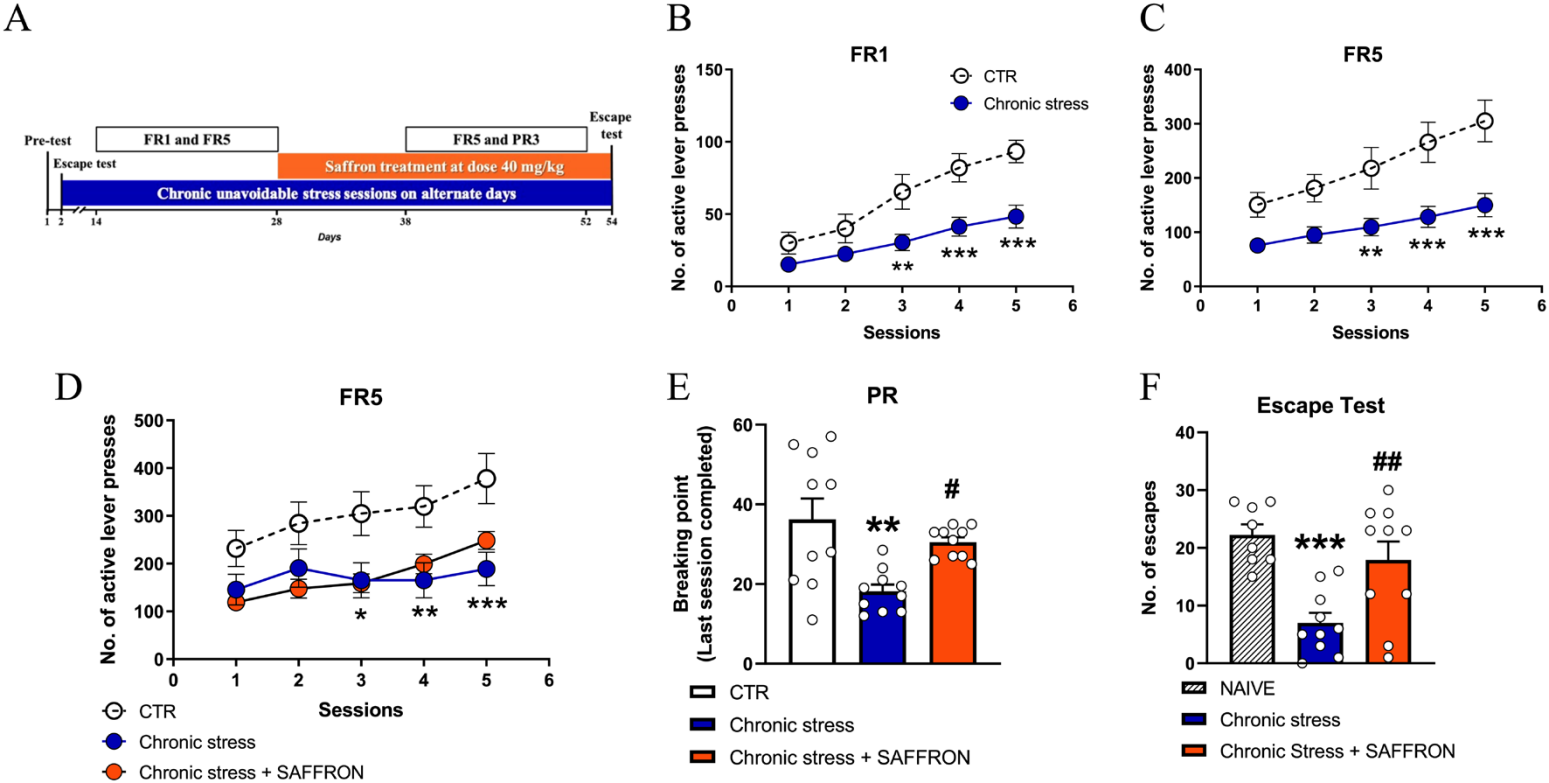
Repeated Saffron administration reinstated the response to positive and aversive stimuli. (A) Outline of the experiment. (B, C) Number of lever presses on the active lever under fixed ratio (FR)1 (B) and FR5 protocols (C). RM Two‐way ANOVA, (B) Stress: *F*_1,28_ = 11.25, *p* < 0.01; Session: *F*_4,112_ = 44.37, *p* < 0.001; Interaction: *F*_4,112_ = 5.118, *p* < 0.001; (C) Stress: *F*_1,28_ = 14.46, *p* < 0.001; Session: *F*_4,112_ = 27.95, *p* < 0.001; Interaction: *F*_4,112_ = 3.826, *p* < 0.01. Post hoc comparisons: **p < 0.01, ***p < 0.001, Sidak's multiple comparisons test. (D) Number of lever presses on the active lever under FR5 after 14–18 days of treatment. RM two‐way ANOVA, Group: *F*_2,27_ = 5.718, *p* < 0.01; Session: *F*_4,108_ = 14.84, *p* < 0.001; interaction: *F*_4,108_ = 2.136, *p* < 0.05. Post hoc comparisons: *p < 0.05, **p < 0.01, ***p < 0.001 Chronic Stress versus CTR group, Sidak's multiple comparisons test. (E) Progressive Ratio (PR) schedule of reinforcement with a step size of 3, One‐way ANOVA, *F*_2,27_ = 7.901, *p* < 0.01. Post hoc comparisons: **p < 0.01 Chronic Stress versus CTR group, ^#^ *p* < 0.05 Chronic Stress + SAFFRON versus Chronic Stress group, Sidak's multiple comparisons test. (F) Number of escapes at the end of saffron treatment, One‐way ANOVA, *F*_2,25_ = 7.245, *p* < 0.001. Post hoc comparisons: ***p < 0.001 Chronic Stress versus NAIVE group, ^##^ *p* < 0.01 Chronic Stress + SAFFRON versus Chronic Stress group, Sidak's multiple comparisons test.

Next, we used an unavoidable Chronic stress protocol to induce in rats a condition of motivational anhedonia, measured in terms of impaired performance in SSA schedules, in order to evaluate the ability of saffron to reinstate operant responding to sucrose (Figure 4A). Rats in the Chronic stress group displayed a clear‐cut impairment in their competence to acquire SSA under FR1 (session 3, *p* < 0.01, sessions 4 and 5, *p* < 0.001, Figure 4B), and FR5 protocols (session 3, *p* < 0.01, sessions 4 and 5, *p* < 0.001, Figure 4C). When the Chronic stress group attained the criterion for appetitive motivation deficit (see Section 2) it was divided into two subgroups that continued the exposure to the chronic stress protocol and received once daily an i.p. administration of saline (Chronic stress) or saffron extract (Chronic stress + SAFFRON) (day 28). Saffron administration for 14–17 days did not restore the competence to operate for sucrose under FR5 schedule, disrupted by stress exposure (Chronic stress vs. CTR group, *p* < 0.05, *p* < 0.01 and *p* < 0.001 at sessions 3, 4 and 5; Figure 4D). However, analysis of the BP values showed that saffron extract reinstated the motivation to press the lever for sucrose (Chronic stress vs. CTR group, *p* < 0.01, Chronic stress + SAFFRON vs. Chronic stress group, *p* < 0.05; Figure 4E). The apparent discrepancy between the results obtained in the FR5 and PR schedules can be likely ascribed to the different length of saffron treatment (e.g., 20–23 days of treatment when the PR schedule was applied, versus 14–17 days of treatment during FR5 training). Thus, we can argue that, for saffron to exert its anti‐anhedonic and antidepressant‐like properties, it is necessary a length of treatment of at least 3 weeks, which in this experiment was reached at the time of exposure to the PR schedule (Figure 4A).

Twenty‐four h after the last PR session, rats in each group were exposed to the escape test to verify their reactivity to avoidable aversive stimuli. Further supporting the positive effect on hyporeactivity to noxious stimuli, saffron treatment was able to restore the escape competence of rats exposed to the chronic unavoidable stress protocol (Chronic stress vs. NAIVE group, *p* < 0.001; Chronic stress vs. Chronic stress + SAFFRON group, *p* < 0.01, Figure 4F).

### 4.5 Repeated Treatment With Saffron Extract Reinstated the DARPP‐32 Phosphorylation Response to a Positive Stimulus

**FIGURE 5:**
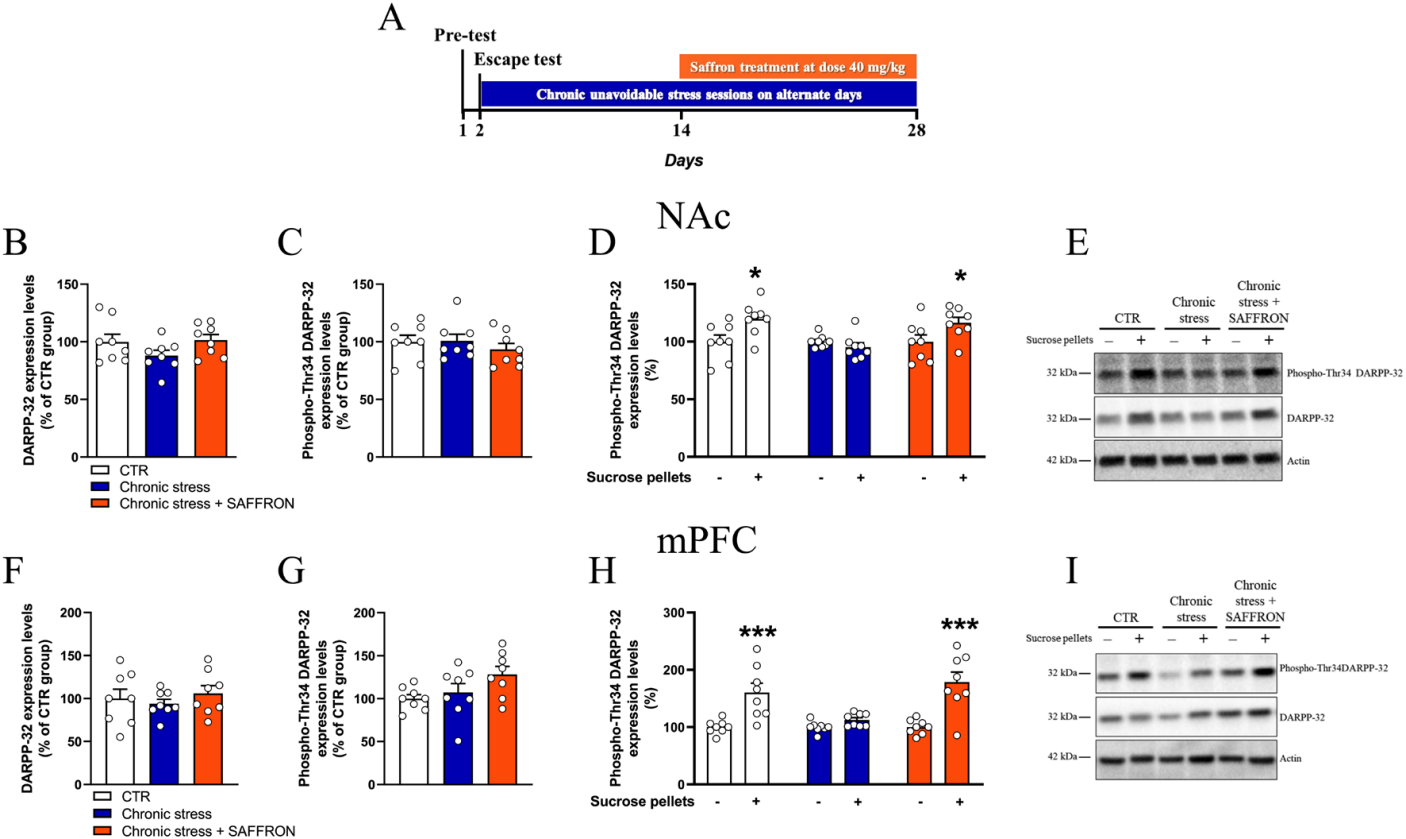
Repeated treatment with saffron reinstated the DARPP‐32 phosphorylation response to a positive stimulus in the nucleus accumbens (B–E) and medial prefrontal cortex (F–I). (A) Outline of the experiment. (B–C) In the NAc, levels of total DARPP‐32 (B, one‐way ANOVA, *F*_2,21_ = 1.836, *p* = 0.1841) and baseline levels of phospho‐Thr34 DARPP‐32 (C, one‐way ANOVA *F*_2,21_ = 0.5386, *p* = 0.5914). (D) Phospho‐Thr34 DARPP‐32 levels in response to consumption of palatable food, expressed as percentage of the baseline values. Two‐way ANOVA, group: *F*_2,42_ = 3.955, *p* < 0.05, palatable stimulus: *F*_1,42_ = 7.493, *p* < 0.01, interaction: *F*_2,42_ = 4.023, *p* < 0.05. Post hoc comparisons: *p < 0.05 compared to no stimulus, Sidak's multiple comparisons test. (E) Representative blots. (F–G) In the mPFC, levels of total DARPP‐32 (F, one‐way ANOVA, *F*_2,21_ = 0.4934, *p* = 0.6174) and baseline levels of phospho‐Thr34 DARPP‐32 (G, One‐way ANOVA, *F*_2,21_ = 3.055, *p* = 0.0684). (H) Phospho‐Thr34 DARPP‐32 response to sucrose, expressed as percentage of the baseline values. Two‐way ANOVA, group: *F*_2,42_ = 5.490, *p* < 0.01, palatable stimulus: *F*_1,42_ = 36.14, *p* < 0.001, interaction: *F*_2,42_ = 5.490, *p* < 0.01. Post hoc comparisons: ***p < 0.001 compared to no stimulus, Sidak's multiple comparisons test. (I) Representative blots. DARPP‐32, dopamine and cAMP‐regulated phosphoprotein, Mr 32 kDa; mPFC, medial prefrontal cortex; NAc, nucleus accumbens.

In order to study whether repeated saffron administration restored the DARPP‐32 response to a reinforcer, disrupted by chronic stress exposure, we examined the phosphorylation pattern of DARPP‐32 in the NAc and mPFC by immunoblotting, at baseline or 30 min after the consumption of sucrose pellets. In the NAc, levels of total DARPP‐32, as well as baseline levels of phospho‐Thr34 DARPP‐32, were similar between experimental groups (Figure 5A,B). The CTR group rats showed an increase in phospho‐Thr34 DARPP‐32 levels, expressed as a percentage of the baseline values, after sucrose consumption (*p* < 0.05), while Chronic stress rats showed a blunted phospho‐Thr34 DARPP‐32 response (Figure 5C). Remarkably, saffron administration reinstated the Thr34‐DARPP‐32 phosphorylation increase in response to sucrose pellet consumption in the Chronic stress + SAFFRON group (*p* < 0.05, Figure 5C). In the mPFC, levels of total DARPP‐32, as well as baseline levels of phospho‐Thr34 DARPP‐32 were similar between experimental groups (Figure 5E,F), phospho‐Thr34 DARPP‐32 levels increased after sucrose consumption in the CTR (*p* < 0.001) and in the Chronic stress + SAFFRON groups (*p* < 0.001), but not in the Chronic stress group (Figure 5G).

### 4.6 Repeated Treatment With Saffron Extract Restored the BDNF/TrkB Signaling Impaired by Chronic Stress Exposure

**FIGURE 6:**
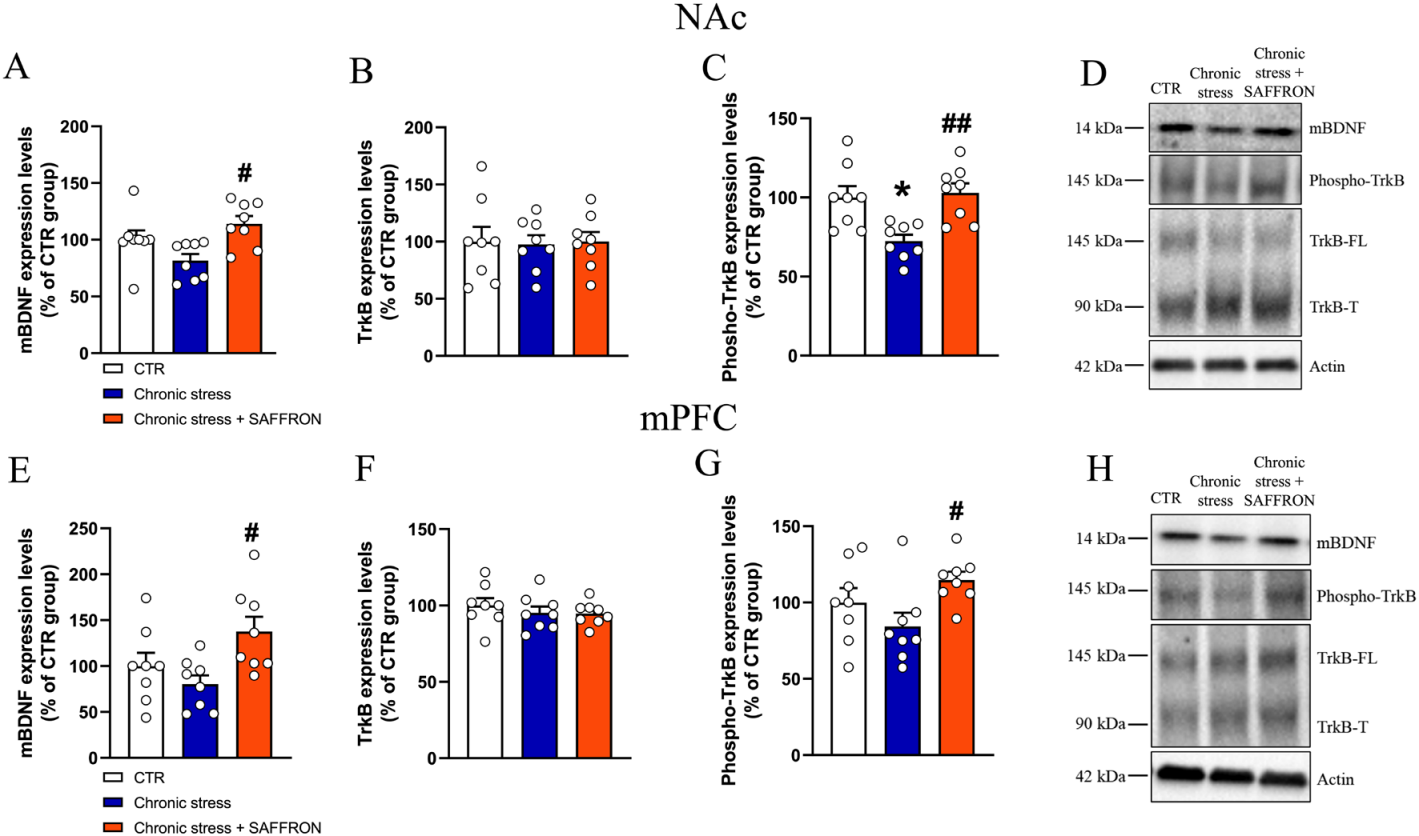
Repeated treatment with saffron extract restored the BDNF/TrkB signaling impaired by Chronic Stress exposure in the nucleus accumbens (A–D) and medial prefrontal cortex (E–H). (A) Levels of mBDNF, One‐way ANOVA *F*_2,21_ = 5.356, *p* < 0.05. Post hoc comparisons: ^#^ *p* < 0.05, Chronic Stress + SAFFRON versus Chronic Stress, Sidak's multiple comparison test. (B) Levels of TrkB, One‐way ANOVA, *F*_2,21_ = 0.02031, *p* = 0.9799. (C) Phospho‐TrkB levels, One‐way ANOVA *F*_2,21_ = 11.94, *p* < 0.001. Post hoc comparisons: *p < 0.05 Chronic Stress versus CTR group, ^##^ *p* < 0.01 Chronic Stress + SAFFRON versus Chronic stress group, Sidak's multiple comparison test. (D) Representative blots. (E) Levels of mBDNF, One‐way ANOVA *F*_2,21_ = 4.534, *p* < 0.05. Post hoc comparisons: ^#^ *p* < 0.05, Chronic Stress + SAFFRON versus Chronic Stress, Sidak's multiple comparison test. (F) TrkB levels, One‐way ANOVA, *F*_2,21_ = 0.5444, *p* = 0.5882. (G) Phospho‐TrkB levels, One‐way ANOVA *F*_2,21_ = 3.498, *p* < 0.05, Post hoc comparisons: ^#^ *p* < 0.05 Chronic Stress + SAFFRON versus Chronic stress group, Sidak's multiple comparison test. (H) Representative blots. mBDNF, mature brain‐derived neurotrophic factor; mPFC, medial prefrontal cortex; NAc, nucleus accumbens; TrkB, tropomyosin receptor kinase B.

In the NAc, mature BDNF (mBDNF) levels were increased after saffron treatment (Chronic stress + SAFFRON vs. Chronic stress, *p* < 0.05, Figure 6A,D), while full‐length TrkB levels were unmodified (Figure 6B,D). Remarkably, significant modifications were detected in the phosphorylation levels of TrkB, namely, a decrease in the Chronic stress compared to the CTR group (*p* < 0.05) and an increase in the Chronic stress + SAFFRON compared to the Chronic stress group (*p* < 0.01, Figure 6C,D). Similar results were obtained in the mPFC, where post hoc comparison demonstrated increased levels of mBDNF and phospho‐TrkB in the Chronic stress + SAFFRON versus the Chronic stress + Saline group (*p* < 0.05 for both comparisons, Figure 6E–H). No changes in the levels of TrkB truncated isoform were observed (Figure S2).

### 4.7 Effect of Saffron as an Add‐On Therapy to Antidepressant Medications in Mild to Moderate Unipolar or Bipolar Depression

**FIGURE 7:**
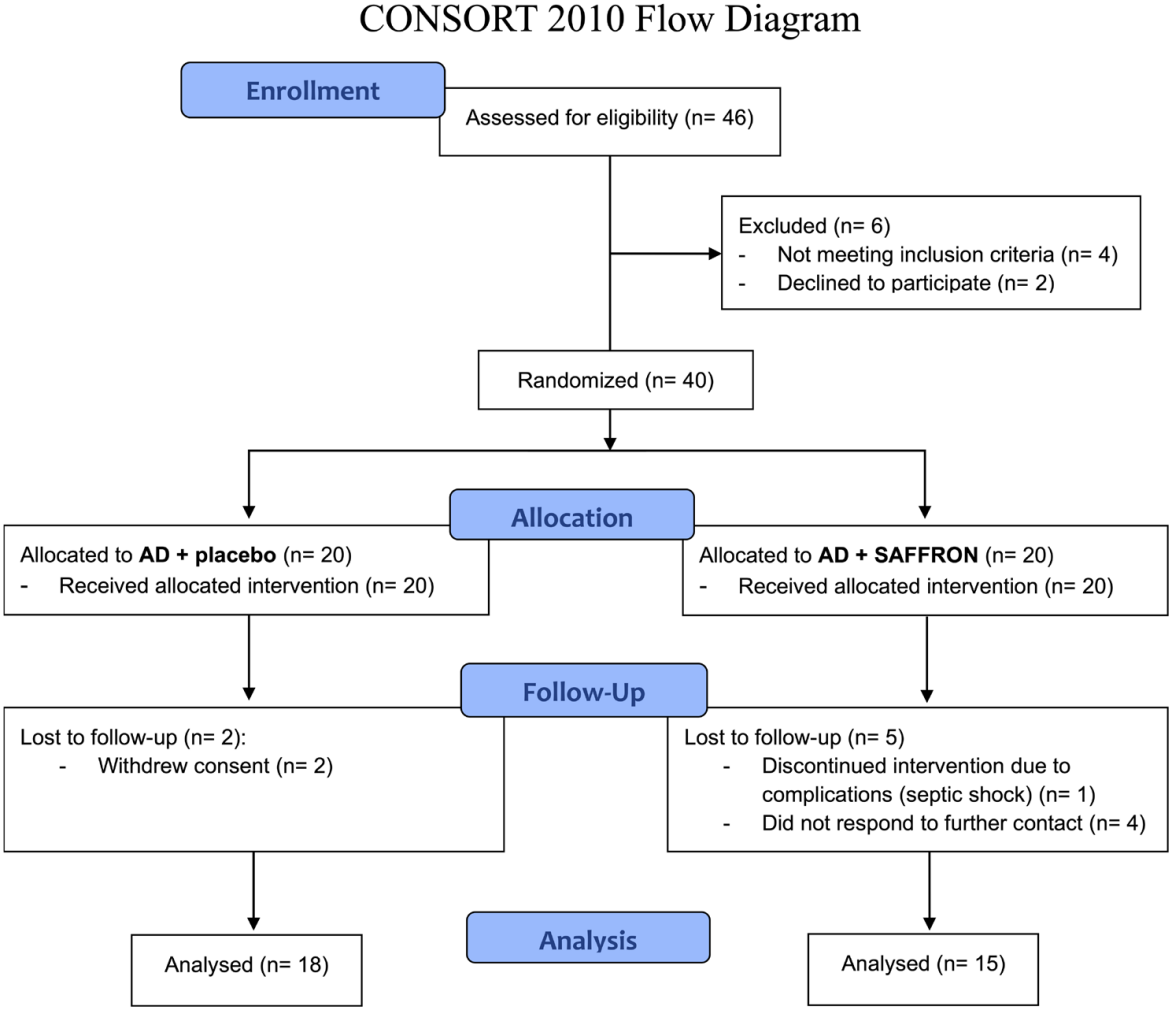
Consort flow diagram. AD + SAFFRON group was administered antidepressant therapy + saffron 30 mg/die.

Forty patients were randomized in the study as shown by the consort flow chart (Figure 7).

Seven participants randomized to treatment were lost to follow‐up (2 in the AD + placebo group and 5 in the AD + SAFFRON group), leaving 18 participants in the AD + placebo group and 15 in the AD + SAFFRON group for final statistical analysis. There were no significant differences in demographics and mean MADRS score at baseline between treatment groups (Table 1).

**TABLE 1:** Patients demographics and clinical characteristics.

Characteristics	Patients randomized (*n* = 40)	Patients completed the study *n* = 33
		AD + placebo: *n* = 18
		AD + SAFFRON: *n* = 15
Age	52.59 ± 2.1 (24–77 years)	AD + placebo: 54.50 ± 2.94 years AD + SAFFRON: 52.67 ± 3.27 years
Sex	Female 28 (70%) Male 12 (30%)	AD + placebo: Female 12; Male 6 AD + SAFFRON: Female 11; Male 4
Diagnosis	32 BD, 8 MDD	AD + placebo: 14 BD, 4 MDD AD + SAFFRON: 13 BD, 2 MDD
Clinical characteristics of treated patients at treatment initiation Basal MADRS score	26.39 ± 1.4	AD + placebo: 27.05 ± 1.80 AD + SAFFRON: 25.60 ± 2.31

Throughout the study, the dosage and formulation of ongoing medications (antidepressants, mood stabilizers, and atypical antipsychotics, as shown in Table S3) remained unchanged for all patients.

**FIGURE 8:**
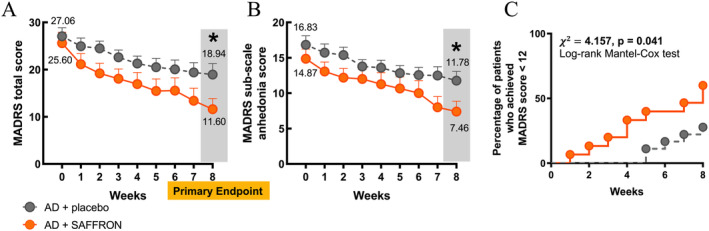
Clinical benefit of saffron add‐on therapy. (A) MADRS total score assessed at enrollment and during the 8‐week treatment. RM two‐way ANOVA, Time: *F*_8,248_ = 30.29, *p* < 0.001; treatment: *F*_1,31_ = 3.081, *p* = 0.089, time × treatment interaction: *F*_8,248_ = 1.602, *p* = 0.124. At the end point (8th week) patients receiving AD + SAFFRON showed a significant reduction in MADRS total score, *p < 0.05, unpaired Student's *t* test. (B) MADRS anhedonia subscale score was evaluated at enrollment and during the 8‐week treatment. RM two‐way ANOVA, time: *F*_8,248_ = 33.03, *p* < 0.001; treatment: *F*_1,31_ = 2.67, *p* = 0.112, treatment × time: *F*_8,848_ = 2.036, *p* < 0.05. (B) at the 8th week saffron induced an improvement in anhedonia symptoms, *p < 0.05, unpaired Student's *t* test. (C) Proportion of patients that achieved a MADRS score < 12, using Kaplan Meier analysis (*Χ* ^*2*^ = 4.157, *p* < 0.05, Log‐Rank Mantel‐Cox test).

Overall, although no significant effect of saffron supplementation on changes in MADRS total score was observed during the study (Figure 8A), saffron induced a small but significant improvement in depressive symptoms and anhedonia scores at the endpoint, when patients receiving AD + SAFFRON showed a significant reduction in MADRS total score [55% from baseline in AD + SAFFRON versus 30% in AD + placebo (*p* < 0.05, independent samples *t* test; Figure 8A)].

Notably, saffron influenced the anhedonia construct (treatment × time interaction: *F*_8,848_ = 2.036, *p* < 0.05); specifically, we observed an improvement in the ANH5 score at the endpoint (*p* < 0.05, unpaired Student's *t* test; Figure 8B). Interestingly, a significant proportion of patients randomized to saffron treatment achieved a MADRS score < 12 at endpoint, which is considered an index of remission (Samalin et al. 2022), compared to subjects receiving placebo (*Χ*_2_ = 4.157, *p* < 0.05, log‐rank Mantel‐Cox test, Figure 8C), as 60% of patients in the AD + SAFFRON group achieved a MADRS score < 12 compared to 27.77% of patients in the AD + placebo group. Consistent with other studies, saffron extract was generally well tolerated, with an increased prevalence of mild gastrointestinal side effects compared to the placebo group.

## 5 Discussion

This study shows that saffron effectively countered stress‐induced motivational anhedonia in rats and had favorable effects as augmentation therapy in unipolar and bipolar subjects reporting mild symptoms of depression. To the best of our knowledge, this is the first evidence that this compound elicits therapeutic effects in the dimension of anhedonia and reward system dysfunction, both in a preclinical study and in a controlled clinical trial.

In the preclinical setting, daily intraperitoneal administration of a Tuscan saffron extract for 3 weeks in a rat model of chronic stress‐induced depression prevented and reverted the condition of escape deficit, that mimics hyporeactivity to negative cues, and reduced motivational anhedonia. The increased reactivity toward aversive stimuli was not related to reduced anxiety or increased locomotor activity.

Recent studies showed that saffron counteracts chronic unavoidable stress‐induced dysfunctions in the hypothalamic–pituitary–adrenal axis in rats, restoring ACTH and corticosterone levels (Kim et al. 2023; Mohammadi et al. 2023). Thus, long‐term saffron administration may counterbalance maladaptive stress responses and promote coping strategies. In this framework, our study focused on the possible activity on motivational anhedonia and using a reinforcement paradigm, we show that saffron blunted the unavoidable stress‐induced impairment in motivation. This positive effect became evident toward the end of the 3rd week of treatment, when saffron restored the motivation to press the lever for sucrose in the PR schedule of reinforcement, which requires higher effort to obtain the reward than the FR schedule. This is the first demonstration of an effect of saffron on motivational anhedonia. Indeed, previous studies using the sucrose preference test (SPT) as a measure of anhedonia and reward dysfunction reported a mild or partial anti‐anhedonic effect but did not explore the possible motivational activity (Orio et al. 2020; Kim et al. 2023). The effects of saffron on SPT were only observed after oral administration and these results have been related to the intestinal metabolism of crocin isomers into crocetin, that seems endowed with a higher antidepressant potential than crocin (Orio et al. 2020). The discrepancies between these and the present results may arise from the differences in the extracts used, as Italian saffron extracts, and in particular the extract used in this study, have a higher content of total crocins than the extracts used in previous studies (Masi et al. 2016). Moreover, studies differ for the definition of anhedonia and the methods used to evaluate it. Indeed, SPT and SSA capture different constructs of reward dysfunction, consummatory versus motivational anhedonia, respectively, which have related but distinct neurobiological underpinnings and respond differently to pharmacological treatments (Scheggi, De Montis, and Gambarana 2018; Kelly, Freeman, and Schumacher 2022). Thus, the present findings support and extend other studies demonstrating the antidepressant‐like activity of saffron and its constituents in mice and rats (Wang et al. 2010; Mohammadi et al. 2023).

The molecular underpinnings of saffron antidepressant effects reported in preclinical and clinical studies need to be further elucidated. Compelling evidence indicates that dysfunctions in dopamine neurotransmission play a critical role in the development of reduced motivation and anhedonia in depression (Salamone and Correa 2012). A condition of motivational anhedonia is indeed associated with reduced dopamine levels in the NAc, blunted dopaminergic response to positive stimuli, impaired dopamine D1 receptor/PKA/DARPP‐32 signaling (Scheggi, De Montis, and Gambarana 2018; Scheggi, Pelliccia, et al. 2018; Cho and Park 2024). This study shows for the first time that in anhedonic rats in which saffron treatment restored the responsiveness to a positive stimulus, also the dopaminergic response to reward was restored. In fact, levels of phospho‐Thr34 DARPP‐32 increased in the NAc and mPFC in response to sucrose pellet consumption. Since PKA‐dependent DARPP‐32 phosphorylation at Thr34 is consequent to dopamine D1 receptor activation, these results suggest that saffron enhanced phasic dopamine release in meso‐cortico‐limbic regions. Supporting this hypothesis, recent studies showed that saffron antidepressant‐like effects in mice are associated with positive modulation of dopaminergic transmission in the striatum and frontal cortex (Monchaux De Oliveira et al. 2021). In prefrontal and striatal regions, dopaminergic transmission, particularly mediated by dopamine D1 receptor activation, modulates reactivity to cues predicting motivationally significant outcomes. Thus, saffron, by modulating dopamine transmission in these brain regions, may restore reactivity and flexibility to reward‐associated cues, and motivation.

BDNF–TrkB signaling is regarded as crucial in the development of a response to antidepressants (Castrén and Antila 2017) and it has been recently demonstrated that antidepressants bind to the transmembrane domain of TrkB, facilitating TrkB membrane retention and thus promoting interaction with BDNF (Casarotto et al. 2021). Although the role of BDNF in depression has been extensively studied in the hippocampus, only recently the activation of BDNF–TrkB pathway in the ventromedial PFC (Liu et al. 2023) and NAc (Li et al. 2023) has been associated with treatments that rescue emotional deficits. Here we used the phosphorylation of TrkB as an index of activation of BDNF signaling. We show that chronic saffron administration increased BDNF levels and enhanced the phosphorylation of full‐length TrkB in the NAc and mPFC of anhedonic rats, suggesting the underlying possible activation of BDNF–TrkB signaling. No changes were observed in TrkB full‐length or truncated isoforms. Previous studies demonstrated that saffron, or its active biological components, increased BDNF expression in the hippocampus, promoting BDNF pathway activation, similar to what was observed after fluoxetine administration (Ghasemi et al. 2015; Mohammadi et al. 2023). While the increased release of BDNF in the hippocampus and mPFC has been classically associated with antidepressant effects, the interpretation of BDNF effects in the NAc is less clear, depending on the nature of stress used to induce a depressive‐like phenotype, pharmacological manipulations and subregion analyzed (e.g., shell and core of NAc) (Berton et al. 2006; Shirayama et al. 2015; Wook Koo et al. 2016). Our results highlight the relevance of BDNF signaling in meso‐cortico‐limbic regions, the NAc and mPFC, and are consistent with the hypothesis that BDNF is a mediator of neuroplastic changes underlying the attenuation of depressive symptoms (Castrén and Antila 2017). Further studies will assess the contribution of BDNF–TrkB signaling in the mechanism of action of saffron, taking advantage of TrkB antagonists, like ANA‐12, and investigate which signaling cascades downstream of TrkB are activated by long‐term saffron administration.

A limitation of this study is that we used only male rats. Indeed, our model of depression induced by chronic stress is based on the learned helplessness (LH) model where rats exposed to inescapable stressors develop a condition of escape deficit (Gambarana et al. 2001). Previous studies showed that while most male rats exposed to uncontrollable stressors develop LH, most female rats learn to escape, irrespective of whether they had been exposed to controllable or uncontrollable stressors, that differently affect the male and female behavior (Dalla et al. 2008; Baratta et al. 2019). Therefore, we considered that before testing a new treatment on both sexes, the development of standardized protocols is needed, in order to study the role of sex influences on LH occurrence and further our understanding of the neurobiological factors that influence stress outcomes in both sexes (Gencturk and Unal 2024).

Anhedonia is highly prevalent among individuals with mood disorders. In the context of unipolar and bipolar depression, depressive episodes show a similar clinical presentation, suggesting that they may share some neurobiological substrates, particularly a common dysfunction of reward processing and dysregulation of dopaminergic activity (Satterthwaite et al. 2015). Thus, since the serotonergic and dopaminergic dysregulation in bipolar depression overlaps with that observed in unipolar depression (Fagiolini et al. 2023) and the management of bipolar depression often mirrors treatment approaches used for unipolar depression, we proposed that saffron antidepressant properties could extend to bipolar depression (Shafiee et al. 2018). Here, with this pilot study, we tested the hypothesis that saffron extract may counteract the abnormal processing of emotional stimuli and therefore may be useful as adjunctive treatment in patients with unipolar or bipolar depression who had not completely responded to their ongoing antidepressant treatment. The strategy to use saffron as add‐on therapy in depressed bipolar patients despite the absence of bipolar‐specific supporting data was based on the consideration that (i) psychiatric treatments often require clinical judgment and the extrapolation of evidence from related conditions, since preclinical models may not fully capture the complexity of mood disorders; (ii) several psychiatric medications have been successfully repurposed for BD without initial preclinical data specific to bipolar depression (Goodwin et al. 2016; McElroy et al. 2010); and (iii) many medications are prescribed across mood disorders, and evidence from unipolar depression studies often informs treatment decisions for bipolar depression, particularly when there are overlapping symptoms such as depressed mood, fatigue, and insomnia (Malhi et al. 2015).

The analysis of the MADRS total score at the end of the 8‐week study showed a global improvement in depressive symptoms with a clinically relevant 7‐point improvement in the MADRS total score at the endpoint (Montgomery 2008) and, remarkably, the analysis of the MADRS anhedonia subscale demonstrated a reduction in the ANH5 anhedonia score, in agreement with our preclinical data. These results are in agreement with previous clinical studies that showed a similar efficacy of saffron extracts compared with that of antidepressant drugs, such as fluoxetine, imipramine or citalopram, with minor side effects (Akhondzadeh et al. 2004, 2005; Noorbala et al. 2005; Shahmansouri et al. 2014). Other clinical studies using saffron extracts or the crocin component as “add‐on therapy” have shown an improvement in clinical response to various antidepressants (Lopresti et al. 2019; Hausenblas et al. 2015; Talaei et al. 2015).

This single‐center, randomized pilot clinical trial presents some limitations. The heterogeneity in concurrent psychopharmacological therapies at enrollment and during the study may have introduced confounding variables, making it challenging to isolate the specific effects of saffron augmentation. Additionally, the small sample size and short follow‐up duration prevented us from assessing long‐term responses, possible sustained activity, and potential long‐term side effects of saffron.

In light of the small sample size, this pilot trial was focused primarily on clinical outcomes and blood samples for biomarkers were not collected. Indeed, while we acknowledge this limitation and the importance of biomarker data as predictors of response to treatment and prognosis, the identification and validation of biomarkers in psychiatry has been, and it still is, challenging (Malik et al. 2021; Trivedi et al. 2016). Thus, based on the results of this first trial, future larger clinical studies should also plan to analyze possible relevant biomarkers, such as serum BDNF and cortisol levels.

Nevertheless, this study provides valuable insights for its focus on the anti‐anhedonic efficacy of saffron augmentation strategy in unipolar or bipolar depression and in a validated animal model of motivational anhedonia. Moreover, the extract used, and geographical location add some relevant regional diversity to the available clinical data, since evidence of publication bias and lack of regional diversity were underscored as limitations of the available saffron studies (Tóth et al. 2019).

## Author Contributions

**Eleonora Corridori:** investigation, methodology, visualization. **Sara Salviati:** investigation, methodology. **Maria Graziella Demontis:** conceptualization, funding acquisition, writing – review and editing. **Pamela Vignolini:** formal analysis, methodology. **Chiara Vita:** funding acquisition. **Andrea Fagiolini:** supervision, writing – review and editing. **Alessandro Cuomo:** formal analysis, investigation. **Pietro Carmellini:** investigation. **Carla Gambarana:** conceptualization, supervision, writing – original draft, writing – review and editing. **Simona Scheggi:** conceptualization, formal analysis, visualization, writing – original draft, writing – review and editing.

## Conflicts of Interest

The authors declare no conflicts of interest.

## Supporting information


Data S1.

## Data Availability

The data that support the findings of this study are available from the corresponding author upon reasonable request.

## References

[ptr8424-bib-0001] Akhondzadeh, S. , H. Fallah‐Pour , K. Afkham , A. H. Jamshidi , and F. Khalighi‐Cigaroudi . 2004. “Comparison of *Crocus sativus* L. and Imipramine in the Treatment of Mild to Moderate Depression: A Pilot Double‐Blind Randomized Trial [ISRCTN45683816].” BMC Complementary and Alternative Medicine 4: 12. 10.1186/1472-6882-4-12. 15341662 PMC517724

[ptr8424-bib-0002] Akhondzadeh, S. , N. Tahmacebi‐Pour , A. A. Noorbala , et al. 2005. “ *Crocus sativus* L. in the Treatment of Mild to Moderate Depression: A Double‐Blind, Randomized and Placebo‐Controlled Trial.” Phytotherapy Research 19, no. 2: 148–151. 10.1002/ptr.1647. 15852492

[ptr8424-bib-0003] Baratta, M. V. , T. M. Gruene , S. D. Dolzani , L. E. Chun , S. F. Maier , and R. M. Shansky . 2019. “Controllable Stress Elicits Circuit‐Specific Patterns of Prefrontal Plasticity in Males, but Not Females.” Brain Structure & Function 224, no. 5: 1831–1843. 10.1007/s00429-019-01875-z. 31028464 PMC6565440

[ptr8424-bib-0004] Berton, O. , C. A. McClung , R. J. Dileone , et al. 2006. “Essential Role of BDNF in the Mesolimbic Dopamine Pathway in Social Defeat Stress.” Science 311, no. 5762: 864–868. 10.1126/science.1120972. 16469931

[ptr8424-bib-0005] Bostan, H. B. , S. Mehri , and H. Hosseinzadeh . 2017. “Toxicology Effects of Saffron and Its Constituents: A Review.” Iranian Journal of Basic Medical Sciences 20, no. 2: 110–121. 10.22038/ijbms.2017.8230. 28293386 PMC5339650

[ptr8424-bib-0006] Carrellas, N. W. , J. Biederman , and M. Uchida . 2017. “How Prevalent and Morbid Are Subthreshold Manifestations of Major Depression in Adolescents? A Literature Review.” Journal of Affective Disorders 210: 166–173. 10.1016/j.jad.2016.12.037. 28049101

[ptr8424-bib-0007] Casarotto, P. C. , M. Girych , S. M. Fred , et al. 2021. “Antidepressant Drugs Act by Directly Binding to TRKB Neurotrophin Receptors.” Cell 184, no. 5: 1299–1313.e19. 10.1016/j.cell.2021.01.034. 33606976 PMC7938888

[ptr8424-bib-0008] Castrén, E. , and H. Antila . 2017. “Neuronal Plasticity and Neurotrophic Factors in Drug Responses.” Molecular Psychiatry 22, no. 8: 1085–1095. 10.1038/mp.2017.61. 28397840 PMC5510719

[ptr8424-bib-0009] Cho, H. , and Y. Park . 2024. “Synergistic Antidepressant‐Like Effects of Biotics and n‐3 Polyunsaturated Fatty Acids on Dopaminergic Pathway Through the Brain‐Gut Axis in Rats Exposed to Chronic Mild Stress.” Probiotics and Antimicrobial Proteins. Advance online publication. 10.1007/s12602-024-10332-1. 39243350

[ptr8424-bib-0010] Dalla, C. , C. Edgecomb , A. S. Whetstone , and T. J. Shors . 2008. “Females Do Not Express Learned Helplessness Like Males Do.” Neuropsychopharmacology 33, no. 7: 1559–1569. 10.1038/sj.npp.1301533. 17712351

[ptr8424-bib-0011] Dev, D. A. , G. H. Le , A. T. H. Kwan , et al. 2024. “Comparing Suicide Completion Rates in Bipolar I Versus Bipolar II Disorder: A Systematic Review and Meta‐Analysis.” Journal of Affective Disorders 361: 480–488. 10.1016/j.jad.2024.06.045. 38901691

[ptr8424-bib-0012] Enkavi, G. , M. Girych , R. Moliner , I. Vattulainen , and E. Castrén . 2024. “TrkB Transmembrane Domain: Bridging Structural Understanding With Therapeutic Strategy.” Trends in Biochemical Sciences 49, no. 5: 445–456. 10.1016/j.tibs.2024.02.001. 38433044

[ptr8424-bib-0013] Ettehadi, H. , S. N. Mojabi , M. Ranjbaran , et al. 2013. “Aqueous Extract of Saffron ( *Crocus sativus* ) Increases Brain Dopamine and Glutamate Concentrations in Rats.” Journal of Behavioral and Brain Science 3: 315–319. 10.4236/jbbs.2013.33031.

[ptr8424-bib-0014] Fagiolini, A. , A. González‐Pinto , K. W. Miskowiak , P. Morgado , A. H. Young , and E. Vieta . 2023. “Role of Trazodone in Treatment of Major Depressive Disorder: An Update.” Annals of General Psychiatry 22, no. 1: 32. 10.1186/s12991-023-00465-y. 37660092 PMC10474647

[ptr8424-bib-0015] Fergusson, D. M. , L. J. Horwood , E. M. Ridder , and A. L. Beautrais . 2005. “Subthreshold Depression in Adolescence and Mental Health Outcomes in Adulthood.” Archives of General Psychiatry 62, no. 1: 66–72. 10.1001/archpsyc.62.1.66. 15630074

[ptr8424-bib-0016] Gambarana, C. , S. Scheggi , A. Tagliamonte , P. Tolu , and M. G. De Montis . 2001. “Animal Models for the Study of Antidepressant Activity.” Brain Research. Brain Research Protocols 7, no. 1: 11–20. 10.1016/s1385-299x(00)00056-8. 11275519

[ptr8424-bib-0017] Gencturk, S. , and G. Unal . 2024. “Rodent Tests of Depression and Anxiety: Construct Validity and Translational Relevance.” Cognitive, Affective, & Behavioral Neuroscience 24, no. 2: 191–224. 10.3758/s13415-024-01171-2. PMC11039509 38413466

[ptr8424-bib-0018] Ghadrdoost, B. , A. A. Vafaei , A. Rashidy‐Pour , et al. 2011. “Protective Effects of Saffron Extract and Its Active Constituent Crocin Against Oxidative Stress and Spatial Learning and Memory Deficits Induced by Chronic Stress in Rats.” European Journal of Pharmacology 667, no. 1–3: 222–229. 10.1016/j.ejphar.2011.05.012. 21616066

[ptr8424-bib-0019] Ghasemi, T. , K. Abnous , F. Vahdati , S. Mehri , B. M. Razavi , and H. Hosseinzadeh . 2015. “Antidepressant Effect of *Crocus sativus* Aqueous Extract and Its Effect on CREB, BDNF, and VGF Transcript and Protein Levels in Rat Hippocampus.” Drug Research 65, no. 7: 337–343. 10.1055/s-0034-1371876. 24696423

[ptr8424-bib-0020] Goodwin, G. M. , P. M. Haddad , I. N. Ferrier , et al. 2016. “Evidence‐Based Guidelines for Treating Bipolar Disorder: Revised Third Edition Recommendations From the British Association for Psychopharmacology.” Journal of Psychopharmacology 30, no. 6: 495–553. 10.1177/0269881116636545. 26979387 PMC4922419

[ptr8424-bib-0021] Harada, K. , M. Aota , T. Inoue , et al. 2006. “Anxiolytic Activity of a Novel Potent Serotonin 5‐HT2C Receptor Antagonist FR260010: A Comparison With Diazepam and Buspirone.” European Journal of Pharmacology 553, no. 1–3: 171–184. 10.1016/j.ejphar.2006.09.042. 17074317

[ptr8424-bib-0022] Hausenblas, H. A. , K. Heekin , H. L. Mutchie , and S. Anton . 2015. “A Systematic Review of Randomized Controlled Trials Examining the Effectiveness of Saffron ( *Crocus sativus* L.) on Psychological and Behavioral Outcomes.” Journal of Integrative Medicine 13, no. 4: 231–240. 10.1016/S2095-4964(15)60176-5. 26165367 PMC5747362

[ptr8424-bib-0023] Kell, G. , A. Rao , G. Beccaria , P. Clayton , A. M. Inarejos‐García , and M. Prodanov . 2017. “Affron a Novel Saffron Extract ( *Crocus sativus* L.) Improves Mood in Healthy Adults Over 4 Weeks in a Double‐Blind, Parallel, Randomized, Placebo‐Controlled Clinical Trial.” Complementary Therapies in Medicine 33: 58–64. 10.1016/j.ctim.2017.06.001. 28735826

[ptr8424-bib-0024] Kelly, C. A. , K. B. Freeman , and J. A. Schumacher . 2022. “Treatment‐Resistant Depression With Anhedonia: Integrating Clinical and Preclinical Approaches to Investigate Distinct Phenotypes.” Neuroscience and Biobehavioral Reviews 136: 104578. 10.1016/j.neubiorev.2022.104578. 35176319

[ptr8424-bib-0025] Kim, C. Y. , K. Ko , S. H. Choi , et al. 2023. “Effects of Saffron Extract (Affron) With 100 Mg/Kg and 200 Mg/Kg on Hypothalamic‐Pituitary‐Adrenal Axis and Stress Resilience in Chronic Mild Stress‐Induced Depression in Wistar Rats.” Nutrients 15, no. 23: 4855. 10.3390/nu15234855. 38068714 PMC10707924

[ptr8424-bib-0026] Kupka, R. W. , L. L. Altshuler , W. A. Nolen , et al. 2007. “Three Times More Days Depressed Than Manic or Hypomanic in Both Bipolar I and Bipolar II Disorder.” Bipolar Disorders 9, no. 5: 531–535. 10.1111/j.1399-5618.2007.00467.x. 17680925

[ptr8424-bib-0027] Li, S. J. , Y. C. Lo , H. Y. Tseng , et al. 2023. “Nucleus Accumbens Deep Brain Stimulation Improves Depressive‐Like Behaviors Through BDNF‐Mediated Alterations in Brain Functional Connectivity of Dopaminergic Pathway.” Neurobiology of Stress 26: 100566. 10.1016/j.ynstr.2023.100566. 37664874 PMC10474237

[ptr8424-bib-0028] Lindahl, J. , M. Asp , D. Ståhl , et al. 2023. “Add‐On Pramipexole for Anhedonic Depression: Study Protocol for a Randomised Controlled Trial and Open‐Label Follow‐Up in Lund, Sweden.” BMJ Open 13, no. 11: e076900. 10.1136/bmjopen-2023-076900. PMC10689415 38035737

[ptr8424-bib-0029] Liu, F. , S. Huang , D. Guo , X. Li , and Y. Han . 2023. “Deep Brain Stimulation of Ventromedial Prefrontal Cortex Reverses Depressive‐Like Behaviors via BDNF/TrkB Signaling Pathway in Rats.” Life Sciences 334: 122222. 10.1016/j.lfs.2023.122222. 38084673

[ptr8424-bib-0030] Lopresti, A. L. , and P. D. Drummond . 2014. “Saffron ( *Crocus sativus* ) for Depression: A Systematic Review of Clinical Studies and Examination of Underlying Antidepressant Mechanisms of Action.” Human Psychopharmacology 29, no. 6: 517–527. 10.1002/hup.2434. 25384672

[ptr8424-bib-0031] Lopresti, A. L. , S. J. Smith , S. D. Hood , and P. D. Drummond . 2019. “Efficacy of a Standardised Saffron Extract (Affron) as an Add‐On to Antidepressant Medication for the Treatment of Persistent Depressive Symptoms in Adults: A Randomised, Double‐Blind, Placebo‐Controlled Study.” Journal of Psychopharmacology 33, no. 11: 1415–1427. 10.1177/0269881119867703. 31475623

[ptr8424-bib-0032] Machado‐Vieira, R. , A. C. Courtes , C. A. Zarate Jr. , I. D. Henter , and H. K. Manji . 2023. “Non‐Canonical Pathways in the Pathophysiology and Therapeutics of Bipolar Disorder.” Frontiers in Neuroscience 17: 1228455. 10.3389/fnins.2023.1228455. 37592949 PMC10427509

[ptr8424-bib-0033] Malhi, G. S. , D. Bassett , P. Boyce , et al. 2015. “Royal Australian and New Zealand College of Psychiatrists Clinical Practice Guidelines for Mood Disorders.” Australian and New Zealand Journal of Psychiatry 49, no. 12: 1087–1206. 10.1177/0004867415617657. 26643054

[ptr8424-bib-0034] Malik, S. , R. Singh , G. Arora , A. Dangol , and S. Goyal . 2021. “Biomarkers of Major Depressive Disorder: Knowing Is Half the Battle.” Clinical Psychopharmacology and Neuroscience 19, no. 1: 12–25. 10.9758/cpn.2021.19.1.12. 33508785 PMC7851463

[ptr8424-bib-0035] Masi, E. , C. Taiti , D. Heimler , P. Vignolini , A. Romani , and S. Mancuso . 2016. “PTR‐TOF‐MS and HPLC Analysis in the Characterization of Saffron ( *Crocus sativus* L.) From Italy and Iran.” Food Chemistry 192: 75–81. 10.1016/j.foodchem.2015.06.090. 26304322

[ptr8424-bib-0036] McElroy, S. L. , R. H. Weisler , W. Chang , et al. 2010. “A Double‐Blind, Placebo‐Controlled Study of Quetiapine and Paroxetine as Monotherapy in Adults With Bipolar Depression (EMBOLDEN II).” Journal of Clinical Psychiatry 71, no. 2: 163–174. 10.4088/JCP.08m04942gre. 20122366

[ptr8424-bib-0037] McIntyre, R. S. , H. Loft , and M. C. Christensen . 2021. “Efficacy of Vortioxetine on Anhedonia: Results From a Pooled Analysis of Short‐Term Studies in Patients With Major Depressive Disorder.” Neuropsychiatric Disease and Treatment 17: 575–585. 10.2147/NDT.S296451. 33654400 PMC7910099

[ptr8424-bib-0038] Minichiello, L. 2009. “TrkB Signalling Pathways in LTP and Learning.” Nature Reviews. Neuroscience 10, no. 12: 850–860. 10.1038/nrn2738. 19927149

[ptr8424-bib-0039] Mohammadi, S. , M. Naseri , N. Faridi , et al. 2023. “Saffron Carotenoids Reversed the UCMS‐Induced Depression and Anxiety in Rats: Behavioral and Biochemical Parameters, and Hippocampal BDNF/ERK/CREB and NR2B Signaling Markers.” Phytomedicine 119: 154989. 10.1016/j.phymed.2023.154989. 37506574

[ptr8424-bib-0040] Monchaux De Oliveira, C. , L. Pourtau , S. Vancassel , et al. 2021. “Saffron Extract‐Induced Improvement of Depressive‐Like Behavior in Mice Is Associated With Modulation of Monoaminergic Neurotransmission.” Nutrients 13, no. 3: 904. 10.3390/nu13030904. 33799507 PMC8001199

[ptr8424-bib-0041] Montgomery, S. 2008. “Clinically Relevant Outcomes Measures in Major Depressive Disorder.” International Journal of Neuropsychopharmacology 11, no. 1: 318.

[ptr8424-bib-0042] Naber, D. , and M. Bullinger . 2018. “Should Antidepressants Be Used in Minor Depression?” Dialogues in Clinical Neuroscience 20, no. 3: 223–228. 10.31887/DCNS.2018.20.3/dnaber. 30581292 PMC6296391

[ptr8424-bib-0043] Noorbala, A. A. , S. Akhondzadeh , N. Tahmacebi‐Pour , and A. H. Jamshidi . 2005. “Hydro‐Alcoholic Extract of *Crocus sativus* L. Versus Fluoxetine in the Treatment of Mild to Moderate Depression: A Double‐Blind, Randomized Pilot Trial.” Journal of Ethnopharmacology 97, no. 2: 281–284. 10.1016/j.jep.2004.11.004. 15707766

[ptr8424-bib-0044] Noyes, B. K. , D. P. Munoz , S. Khalid‐Khan , E. Brietzke , and L. Booij . 2022. “Is Subthreshold Depression in Adolescence Clinically Relevant?” Journal of Affective Disorders 309: 123–130. 10.1016/j.jad.2022.04.067. 35429521

[ptr8424-bib-0045] Orio, L. , F. Alen , A. Ballesta , R. Martin , and R. Gomez de Heras . 2020. “Antianhedonic and Antidepressant Effects of Affron, a Standardized Saffron ( *Crocus Sativus* L.) Extract.” Molecules 25, no. 14: 3207. 10.3390/molecules25143207. 32679643 PMC7397008

[ptr8424-bib-0046] Padovan, C. M. , and F. S. Guimarães . 2000. “Restraint‐Induced Hypoactivity in an Elevated Plus‐Maze.” Brazilian Journal of Medical and Biological Research 33, no. 1: 79–83. 10.1590/s0100-879x2000000100011. 10625878

[ptr8424-bib-0047] Paxinos, G. , and C. Watson . 2007. The Rat Brain in Stereotaxic Coordinates. 6th ed. San Diego: Elsevier Academic Press.

[ptr8424-bib-0048] Pellow, S. , P. Chopin , S. E. File , and M. Briley . 1985. “Validation of Open:Closed Arm Entries in an Elevated Plus‐Maze as a Measure of Anxiety in the Rat.” Journal of Neuroscience Methods 14, no. 3: 149–167. 10.1016/0165-0270(85)90031-7. 2864480

[ptr8424-bib-0049] Saha, S. , C. C. W. Lim , D. L. Cannon , et al. 2021. “Co‐Morbidity Between Mood and Anxiety Disorders: A Systematic Review and Meta‐Analysis.” Depression and Anxiety 38, no. 3: 286–306. 10.1002/da.23113. 33225514 PMC7984258

[ptr8424-bib-0050] Salamone, J. D. , and M. Correa . 2012. “The Mysterious Motivational Functions of Mesolimbic Dopamine.” Neuron 76, no. 3: 470–485. 10.1016/j.neuron.2012.10.021. 23141060 PMC4450094

[ptr8424-bib-0051] Samalin, L. , M. Rothärmel , L. Mekaoui , et al. 2022. “Esketamine Nasal Spray in Patients With Treatment‐Resistant Depression: The Real‐World Experience in the French Cohort Early‐Access Programme.” International Journal of Psychiatry in Clinical Practice 26, no. 4: 352–362. 35174754 10.1080/13651501.2022.2030757

[ptr8424-bib-0052] Satterthwaite, T. D. , J. W. Kable , L. Vandekar , et al. 2015. “Common and Dissociable Dysfunction of the Reward System in Bipolar and Unipolar Depression.” Neuropsychopharmacology 40, no. 9: 2258–2268. 10.1038/npp.2015.75. 25767910 PMC4613620

[ptr8424-bib-0053] Scheggi, S. , M. G. De Montis , and C. Gambarana . 2018. “DARPP‐32 in the Orchestration of Responses to Positive Natural Stimuli.” Journal of Neurochemistry 147, no. 4: 439–453. 10.1111/jnc.14558. 30043390

[ptr8424-bib-0054] Scheggi, S. , F. Guzzi , G. Braccagni , M. G. De Montis , M. Parenti , and C. Gambarana . 2020. “Targeting PPARα in the Rat Valproic Acid Model of Autism: Focus on Social Motivational Impairment and Sex‐Related Differences.” Molecular Autism 11, no. 1: 62. 10.1186/s13229-020-00358-x. 32718349 PMC7385875

[ptr8424-bib-0055] Scheggi, S. , T. Pelliccia , C. Gambarana , and M. G. De Montis . 2018. “Aripiprazole Relieves Motivational Anhedonia in Rats.” Journal of Affective Disorders 227: 192–197. 10.1016/j.jad.2017.10.032. 29100151

[ptr8424-bib-0056] Shafiee, M. , S. Arekhi , A. Omranzadeh , and A. Sahebkar . 2018. “Saffron in the Treatment of Depression, Anxiety and Other Mental Disorders: Current Evidence and Potential Mechanisms of Action.” Journal of Affective Disorders 227: 330–337. 10.1016/j.jad.2017.11.020. 29136602

[ptr8424-bib-0057] Shahmansouri, N. , M. Farokhnia , S. H. Abbasi , et al. 2014. “A Randomized, Double‐Blind, Clinical Trial Comparing the Efficacy and Safety of *Crocus sativus* L. With Fluoxetine for Improving Mild to Moderate Depression in Post Percutaneous Coronary Intervention Patients.” Journal of Affective Disorders 155: 216–222. 10.1016/j.jad.2013.11.003. 24289892

[ptr8424-bib-0058] Shirayama, Y. , C. Yang , J. C. Zhang , Q. Ren , W. Yao , and K. Hashimoto . 2015. “Alterations in Brain‐Derived Neurotrophic Factor (BDNF) and Its Precursor proBDNF in the Brain Regions of a Learned Helplessness Rat Model and the Antidepressant Effects of a TrkB Agonist and Antagonist.” European Neuropsychopharmacology 25, no. 12: 2449–2458. 10.1016/j.euroneuro.2015.09.002. 26419294

[ptr8424-bib-0059] Talaei, A. , M. Hassanpour Moghadam , S. A. Sajadi Tabassi , and S. A. Mohajeri . 2015. “Crocin, the Main Active Saffron Constituent, as an Adjunctive Treatment in Major Depressive Disorder: A Randomized, Double‐Blind, Placebo‐Controlled, Pilot Clinical Trial.” Journal of Affective Disorders 174: 51–56. 10.1016/j.jad.2014.11.035. 25484177

[ptr8424-bib-0060] Tóth, B. , P. Hegyi , T. Lantos , et al. 2019. “The Efficacy of Saffron in the Treatment of Mild to Moderate Depression: A Meta‐Analysis.” Planta Medica 85, no. 1: 24–31. 10.1055/a-0660-9565. 30036891

[ptr8424-bib-0061] Treadway, M. T. , J. A. Cooper , and A. H. Miller . 2019. “Can't or Won't? Immunometabolic Constraints on Dopaminergic Drive.” Trends in Cognitive Sciences 23, no. 5: 435–448. 10.1016/j.tics.2019.03.003. 30948204 PMC6839942

[ptr8424-bib-0062] Trivedi, M. H. , P. J. McGrath , M. Fava , et al. 2016. “Establishing Moderators and Biosignatures of Antidepressant Response in Clinical Care (EMBARC): Rationale and Design.” Journal of Psychiatric Research 78: 11–23. 10.1016/j.jpsychires.2016.03.001. 27038550 PMC6100771

[ptr8424-bib-0063] Vanderlind, W. M. , Y. Millgram , A. R. Baskin‐Sommers , M. S. Clark , and J. Joormann . 2020. “Understanding Positive Emotion Deficits in Depression: From Emotion Preferences to Emotion Regulation.” Clinical Psychology Review 76: 101826. 10.1016/j.cpr.2020.101826. 32058881

[ptr8424-bib-0064] Vignolini, P. , D. Heimler , P. Pinelli , F. Ieri , A. Sciullo , and A. Romani . 2008. “Characterization of By‐Products of Saffron ( *Crocus Sativus* L.) Production.” Natural Product Communications 3, no. 12: 1959–1962. 10.1177/1934578X0800301203.

[ptr8424-bib-0065] Wang, Y. , T. Han , Y. Zhu , et al. 2010. “Antidepressant Properties of Bioactive Fractions From the Extract of *Crocus sativus* L.” Journal of Natural Medicines 64, no. 1: 24–30. 10.1007/s11418-009-0360-6. 19787421

[ptr8424-bib-0066] Wook Koo, J. , B. Labonté , O. Engmann , et al. 2016. “Essential Role of Mesolimbic Brain‐Derived Neurotrophic Factor in Chronic Social Stress‐Induced Depressive Behaviors.” Biological Psychiatry 80, no. 6: 469–478. 10.1016/j.biopsych.2015.12.009. 26858215 PMC4909591

