# Supplementary material for: Therapeutic Potential of Saffron Extract in Mild Depression: A Study of Its Role on Anhedonia in Rats and Humans
Source: Phytother Res. 2025 Jan 4;39(3):1277–91. doi: 10.1002/ptr.8424 (PMC11891950; doi:10.1002/ptr.8424)
Supplement: Supplementary file 1 — Data S1. [file PTR-39-1277-s001.pdf]

Table S1. Composition of the saffron and placebo capsules.

	Saffron capsule	***Placebo capsule***
Active ingredient (saffron)	7,5 mg	--
Colloidal silica	2 mg	2 mg
Magnesium stearate	5 mg	5 mg
Microcrystalline cellulose	75,5 mg	75,5 mg
Calcium phosphate	160,0 mg	167,5 mg
White gelatin capsule	100 mg	100 mg

Table S2**.** All statistical results of graphs presented in figures throughout the article.
The results of post-hoc comparisons are reported in the legend of the corresponding figure.
Significant values are shown in red.

A. Statistical details of results presented in Fig. 3

**Fig. 3B**	**One-way ANOVA**	**F (DFn. DFd)**	**P value**	**Partial η²**
Time spent in open arm	*Groups comparison*	*F (3, 36) = 4.136*	*P=0.0128*	*0.2564*
**Fig. 3C**	**One-way ANOVA**	**F (DFn. DFd)**	**P value**	**Partial η²**
Time spent in closed arm	*Groups comparison*	*F (3, 36) = 4.177*	*P=0.0123*	*0.2582*
**Fig. 3D**	**One-way ANOVA**	**F (DFn. DFd)**	**P value**	**Partial η²**
Number of entries	Groups comparison	F (3, 36) = 1.029	P=0.3912	0.0790
**Fig. 3E**	**One-way ANOVA**	**F (DFn. DFd)**	**P value**	**Partial η²**
Number of escapes	*Groups comparison*	*F (3, 36) = 5.020*	*P=0.0052*	*0.2949*

B. Statistical details of results presented in Fig. 4

**Fig. 4B**	**Two-way ANOVA**	**F (DFn. DFd)**	**P value**	**Partial η²**
FR1	*Main effect Stress*	*F (1, 28) = 11.25*	*P=0.0023*	*0.287*
	Main effect Session	*F (4, 112) = 44.37*	*P<0.0001*	*0.613*
	Stress x Session interaction	*F (4, 112) = 5.118*	*P=0.0008*	*0.155*
**Fig. 4C**	**Two-way ANOVA**	**F (DFn. DFd)**	**P value**	**Partial η²**
FR5	*Main effect Stress*	*F (1, 28) = 14.46*	*P=0.0007*	*0.341*
	Main effect Session	*F (4, 112) = 27.95*	*P<0.0001*	*0.500*
	Stress x Session interaction	*F (4, 112) = 3.826*	*P=0.0059*	*0.120*
**Fig. 4D**	**Two-way ANOVA**	**F (DFn. DFd)**	**P value**	**Partial η²**
FR5	*Main effect Group*	*F (2, 27) = 5.718*	*P=0.0085*	*0.298*
	Main effect Session	*F (4, 108) = 14.84*	*P<0.0001*	*0.355*
	Group x Session interaction	*F (8, 108) = 2.136*	*P=0.0383*	*0.137*

**Fig. 4E**	**One-way ANOVA**	**F (DFn. DFd)**	**P value**	**Partial η^2**
PR (BP)	*Groups comparison*	*F (2, 27) = 7.901*	*P=0.0020*	*0.369*
**Fig. 4F**	**One-way ANOVA**	**F (DFn. DFd)**	**P value**	**Partial η^2**
Number of escape	*Groups comparison*	*F (2, 25) = 10.27*	*P=0.0006*	*0.4512*

C. Statistical details of results presented in Fig. 5

	**Fig. 5B**	**One-way ANOVA**	**F (DFn. DFd)**	**P value**	**Partial η^2**
	DARPP-32	Groups comparison	F (2, 21) = 1.836	P=0.1841	0.1488
	**Fig. 5C**	**One-way ANOVA**	**F (DFn. DFd)**	**P value**	**Partial η^2**
**NAc**	PhosphoThr34 DARPP-32	Groups comparison	F (2, 21) = 0.5386	P=0.5914	0.0488
	**Fig. 5D**	**Two-way ANOVA**	**F (DFn. DFd)**	**P value**	**Partial η^2**
		Main effect Group	*F (2, 42) = 3.955*	*P=0.0267*	*0.159*
	PhosphoThr34 DARPP-32	*Main effect Stimulus*	*F (1, 42) = 7.493*	*P=0.0090*	*0.151*
		Group x Stimulus interaction	*F (2, 42) = 4.023*	*P=0.0252*	*0.161*
	**Fig. 5F**	**One-way ANOVA**	**F (DFn. DFd)**	**P value**	**Partial η^2**
	DARPP-32	Groups comparison	F (2, 21) = 0.4934	P=0.6174	0.0449
	**Fig. 5G**	**One-way ANOVA**	**F (DFn. DFd)**	**P value**	**Partial η^2**
**mPFC**	PhosphoThr34 DARPP-32	Groups comparison	F (2, 21) = 3.055	P=0.0684	0.2254
	**Fig. 5H**	**Two-way ANOVA**	**F (DFn. DFd)**	**P value**	**Partial η^2**
		Main effect Group	*F (2, 42) = 5.490*	*P=0.0076*	*0.207*
	PhosphoThr34 DARPP-32	*Main effect Stimulus*	*F (1, 42) = 36.14*	*P<0.0001*	*0.462*
		Group x Stimulus interaction	*F (2, 42) = 5.490*	*P=0.0076*	*0.207*

D. Statistical details of results presented in Fig. 6

	**Fig. 6A**	**One-way ANOVA**	**F (DFn. DFd)**	**P value**	**Partial η^2**
	mBDNF	*Groups comparison*	*F (2, 21) = 5.356*	*P=0.0132*	*0.3378*

**Nac**	**Fig. 6B**	**One-way ANOVA**	**F (DFn. DFd)**	**P value**	**Partial η^2**
	TrkB-FL	Groups comparison	F (2, 21) = 0.0203	P=0.9799	0.0019
	**Fig. 6C**	**One-way ANOVA**	**F (DFn. DFd)**	**P value**	**Partial η^2**
	Phospho-TrKB	*Groups comparison*	*F (2, 21) = 8.121*	*P=0.0024*	*0.4361*
**mPFC**	**Fig. 6E**	**One-way ANOVA**	**F (DFn. DFd)**	**P value**	**Partial η^2**
	mBDNF	*Groups comparison*	*F (2, 21) = 4.534*	*P=0.0231*	*0.3016*
	**Fig. 6F**	**One-way ANOVA**	**F (DFn. DFd)**	**P value**	**Partial η^2**
	TrkB-FL	Groups comparison	F (2, 21) = 0.5444	P=0.5882	0.0493
	**Fig. 6G**	**One-way ANOVA**	**F (DFn. DFd)**	**P value**	**Partial η^2**
	Phospho-TrKB	*Groups comparison*	*F (2, 21) = 3.498*	*P=0.0488*	*0.2499*

E. Statistical details of results presented in Fig. 8

**Fig. 8A**	**Two-way ANOVA**	**F (DFn. DFd)**	**P value**	**Partial η^2**
MADRS total score	Main effect Treatment	F (1, 31) = 2.999	P=0.0933	0.088
	Main effect Time	*F (8, 248) = 30.29*	*P<0.0001*	*0.494*
	Treatment x Time interaction	F (8, 248) = 1.602	P=0.1246	0.049
**Fig. 8B**	**Unpaired t test**	**F (DFn. DFd)**	**P value**	**Partial η^2**
MADRS subscale anhedonia	Main effect Treatment	F (1, 31) = 2.672	P=0.1122	0.079
	Main effect Time	*F (8, 248) = 33.03*	*P<0.0001*	*0.516*
	Treatment x Time interaction	*F (8, 248) = 2.036*	*P=0.0429*	*0.062*
**Fig. 8C**	**Comparison of Survival Curves**	**Chi square**	**P value**	
Survival curve	*Saffron vs Placebo*	*4.157*	*P=0.0415*	

F. Statistical details of results presented in Fig. S1

**Fig. S1.A**	**Two-way ANOVA**	**F (DFn. DFd)**	**P value**	**Partial η^2**
FR1	Main effect Treatment	F (1, 9) = 0.02408	P=0.8801	0.003
	Main effect Session	*F (4, 36) = 10.68*	*P<0.0001*	*0.543*
	Treatment x Session interaction	F (4, 36) = 0.4646	P=0.7612	0.049
**Fig. S1.B**	**Two-way ANOVA**	**F (DFn. DFd)**	**P value**	**Partial η^2**
FR5	Main effect Treatment	F (1, 9) = 0.3582	P=0.5643	0.038
	Main effect Session	*F (4, 36) = 10.15*	*P<0.0001*	*0.530*
	Treatment x Session interaction	F (4, 36) = 0.2042	P=0.9344	0.022
**Fig. S1.C**	**Unpaired t test**	**F (DFn. DFd)**	**P value**	**Partial η^2**
PR (BP)	Saffron vs Saline	F (4, 5) = 1.050	P=0.3747	0.088

G. Statistical details of results presented in Fig. S2

**Nac**	**Fig. S2.A**	**One-way ANOVA**	**F (DFn. DFd)**	**P value**	**Partial η^2**
	TrkB-T	Groups comparison	F (2, 21) = 0.5699	P=0.7999	0.05149
**mPFC**	**Fig. S2.B**	**One-way ANOVA**	**F (DFn. DFd)**	**P value**	**Partial η^2**
	TrkB-T	Groups comparison	F (2, 21) = 2.144	P=0.1421	0.1696

Table S3. List of patients' medications.

Group	Patient	Sex	Age	Diagnosis	Mood Stabilizers	Antidepressants	Antipsychotics
**AD + Placebo**	S05	M	64	MDD	Gabapentin	Sertraline	Aripiprazole, Quetiapine
	S06	F	28	BD	Lithium	Citalopram	Aripiprazole
	S07	M	58	BD	Lithium	Citalopram	Aripiprazole, Quetiapine
	S09	F	56	BD	Valproate, Lamotrigine	--	--
	S13	F	52	BD	Lithium, lamotrigine	Sertraline	Aripiprazole
	S14	F	56	BD	Lithium	Citalopram	Quetiapine
	S15	M	52	MDD	Valproate	--	Quetiapine
	S19	F	41	BD	Lithium, Valproate	Paroxetine, Mirtazapine	Quetiapine
	S22	F	30	MDD	--	Sertraline	--
	S24	F	61	MDD	--	Sertraline	--
	S27	F	67	BD	Valproate, Lamotrigine	Paroxetine	--
	S31	M	64	BD	Lithium	Escitalopram	Quetiapine
	S32	F	54	BD	Valproate	Paroxetine	Olanzapine
	S34	M	63	BD	Lithium, Valproate, Gabapentin	Citalopram	Quetiapine, amisulpiride
	S36	M	54	BD	Valproate, Pregabalin	Sertraline	--
	S38	F	77	BD	Lithium, Valproate	Sertraline	--
	S39	F	61	BD	Litio, Lamotrigine	Sertraline	Brexpiprazole, Olanzapine
	S40	F	43	BD	Lithium, Valproate	Citalopram, Vortioxetine	Lurasidone, Quetiapine
Drop-out	*S28*	*F*	*31*	*BD*	*Lithium, Pregabalin*	*Vortioxetine*	*Aripiprazole, Aloperidol*
Drop-out	*S33*	*F*	*25*	MDD	*--*	*Vortioxetine*	*--*
**AD + SAFFRON**	S01	M	61	BD	Valproate	Sertraline	--
	S03	F	67	BD	Lithium	Mirtazapine	Quetiapine
	S04	M	60	BD	Valproate	Vortioxetine	Olanzapine
	S08	F	65	BD	Lithium	Citalopram	Quetiapine, Aripiprazole
	S10	F	24	BD	Lithium, Pregabalin	Sertraline	Quetiapine
	S16	M	53	MDD	Carbamazepine	Sertraline	--
	S18	F	62	MDD	--	Paroxetine	--
	S20	F	56	BD	Lithium	Sertraline	Aripiprazole
	S21	M	40	BD	Lithium, valproate	--	Quetiapine
	S23	F	52	BD	Lithium, valproate, pregabalin	Sertraline	Olanzapine
	S25	F	41	BD	Lithium, valproate, pregabalin,	Citalopram	Quetiapine
	S26	F	48	BD	Lithium	Citalopram	Quetiapine
	S29	F	41	BD	--	Citalopram	Quetiapine
	S30	F	48	BD	--	Citalopram	Olanzapine
	S37	F	72	BD	Valproate	Sertraline	--
Drop-out	*S02*	*F*	*61*	*BD*	*Lithium, Valproate*	*Paroxetine*	*Quetiapine*
Drop-out	*S11*	*F*	*68*	*BD*	*ND*	*ND*	*ND*
Drop-out	*S12*	*F*	*51*	*BD*	*Valproate*	*Bupropion*	--
Drop-out	*S17*	*M*	*44*	MDD	*ND*	*ND*	*ND*
Drop-out	*S35*	*M*	*ND*	*BD*	*Pregabalin*	*Sertraline*	*Olanzapine*

Fig.S1

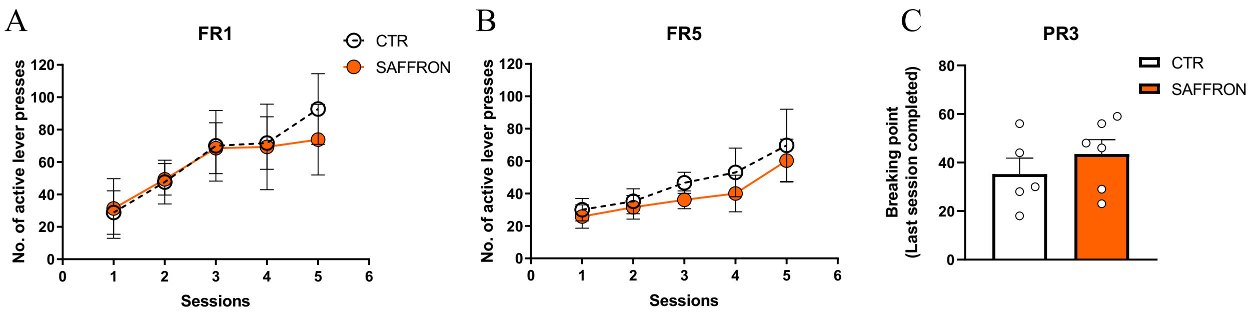

Fig.S1 Effect of a repeated treatment with saffron extract on the acquisition of sucrose self administration (SSA)

(A, B) Number of lever presses on the active lever under Fixed Ratio (FR)1 (B) and FR5 protocols (C) Progressive Ratio (PR) schedule of reinforcement with a step size of 3.

After one week of treatment, saline-treated control rats (n = 5) and saffron-treated rats (n = 6) were trained in SSA protocols (FR1, FR5 and PR3), while continuing treatment. Long-term saffron treatment did not induce gross behavioral modifications and did not affect motor activity or performance in the self-administration protocols, indicating that the extract did not modify cognitive and learning abilities or reward threshold.

Fig.S2

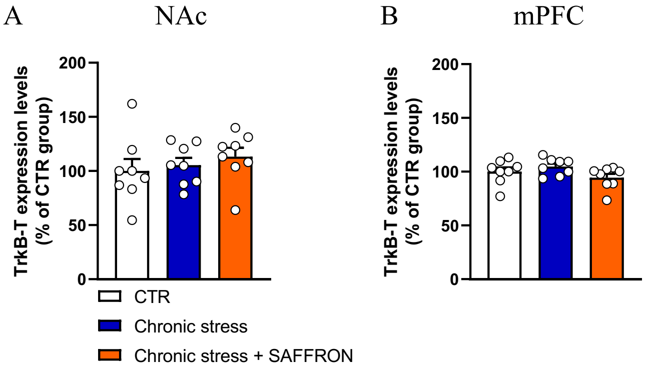

Fig.S2 Repeated treatment with saffron extract on the expression level of the truncated isoform of TrkB.

(A) Levels of truncated TrkB (TrkB-T) in the NAc; (B) Levels of truncated TrkB (TrkB-T) in the mPFC. There were no changes in the levels of TrkB truncated isoform in western blot experiments.

TrkB: Tropomyosin receptor kinase B; NAc: nucleus accumbens; mPFC: medial prefrontal cortex.
